# Combining Quantitative Susceptibility Mapping With the Gray Matter Volume to Predict Neurological Deficits in Patients With Small Artery Occlusion

**DOI:** 10.1002/brb3.70080

**Published:** 2024-10-04

**Authors:** Xuelian Tang, Zhenzhen He, Qian Yang, Tao Yang, Yusheng Yu, Jinan Chen

**Affiliations:** ^1^ Department of Neurology The Affiliated Jiangning Hospital of Nanjing Medical University Nanjing Jiangsu China; ^2^ Department of Radiology The Affiliated Jiangning Hospital of Nanjing Medical University Nanjing Jiangsu China

**Keywords:** gray matter volume | neurological deficits | quantitative susceptibility mapping | small artery occlusion

## Abstract

**Background:**

Currently, there is still a lack of valuable neuroimaging markers to assess the clinical severity of stroke patients with small artery occlusion (SAO). Quantitative susceptibility mapping (QSM) is a quantitative processing method for neuroradiological diagnostics. Gray matter (GM) volume changes in stroke patients are also proved to be associated with neurological deficits. This study aims to explore the predictive value of QSM and GM volume in neurological deficits of patients with SAO.

**Methods:**

As neurological deficits, the National Institutes of Health Stroke Scale (NIHSS) was used. Sixty‐six SAO participants within 24 h of first onset were enrolled and divided into mild and moderate groups based on NIHSS. QSM values of infarct area and GM volume were calculated from magnetic resonance imaging (MRI) data. Two‐sample *t*‐tests were used to compare differences in QSM value and GM volume between the two groups, and the diagnostic efficacy of the combination of QSM value and GM volume was evaluated.

**Results:**

The results revealed both the QSM value and GM volume within the infarct area of the moderate group were lower compared to the mild group. Moderate group exhibited lower GM volume in some specific gyrus compared with mild group in the case of voxel‐wise GM volume on whole‐brain voxel level. The support vector machine (SVM) classifier's analysis showed a high power for the combination of QSM value, GM volume within the infarct area, and voxel‐wise GM volume.

**Conclusion:**

Our research first reported the combination of QSM value, GM volume within the infarct area, and voxel‐wise GM volume could be used to predict neurological impairment of patients with SAO, which provides new insights for further understanding the SAO stroke.

## Graphical abstract

The combination of QSM values, GM volumes displayed a strong ability to differentiate SAO‐MoS from SAO‐MiS, which may provide us new insights to understand the neurological deficits in SAO stroke.

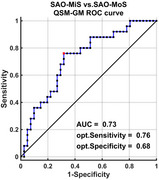

## 1 Introduction

Ischemic stroke is a leading contributor to global disability and mortality (Fan et al. 2023). Small artery occlusion (SAO) accounts for 27.3% of acute ischemic stroke in developing countries (Xin et al. 2019). Approximately 20%–30% of SAO patients experience neurological deterioration within days after stroke onset, even if they have received adequate treatments (Berberich et al. 2020). It is urgent for us to further understand the SAO stroke. Traditionally, computed tomography angiography (CTA) and magnetic resonance angiography (MRA) are routinely performed to evaluate the clinical severity and outcomes of acute stroke patients, whereas they are more sensitive to large‐artery atherosclerosis and cardio embolism subtypes (Czap and Sheth 2021). The National Institutes of Health Stroke Scale (NIHSS) is a common instrument to evaluate the severity of neurological deficits of ischemic stroke. However, NIHSS has certain limitations, such as that it cannot accurately evaluate posterior circulation stroke, and it is also always affected by various artificial factors. It is very necessary for us to find other accurate and reliable methods to predict the severity of neurological deficits in acute stroke patients.

To date, there are still a shortage of suitable neurovascular imaging markers to assess the stroke severity of SAO patients. Susceptibility‐weighted imaging (SWI) is an emerging and noninvasive neuroimaging technique that enhances the magnetic susceptibility differences of various compounds in brain (Haacke et al. 2004). Recently, SWI has been reported to evaluate the vascular structures and consequences of patients with ischemic stroke by enhancing the visualization of small vessels (Liu et al. 2015; Çetinkaya et al. 2023). However, SWI is a kind of qualitative measurement tool that is easily affected by artificial factors. Quantitative susceptibility mapping (QSM) is a quantitative MR imaging technique that is applied to demonstrate the characteristics of various central nervous system diseases (Liu et al. 2015; Zeineddine et al. 2018). A recent study showed that QSM provides highly sensitive and specific biomarkers of completed remyelination in multiple sclerosis (Rahmanzadeh et al. 2022). A report from monkey brains suggested that QSM may provide complementary information for understanding the neuropathology of the brain tissue following stroke and predicting stroke outcomes (Meng, Li, and Zhang 2023). Another study showed susceptibility measurements of thrombi by QSM may helped predict cardioembolic stroke in patients with acute middle cerebral artery occlusion (Chen, Zhang et al. 2022a), whereas the application of QSM in SAO stroke is still unexplored.

Kataike et al. (2024) reported iron concentration in infarct tissue after successful endovascular reperfusion increases compared to that in healthy tissue as measured by QSM. The changes of iron‐level‐related QSM value were related to the deductions of gray matter (GM) volume (Chai et al. 2015). Moreover, some studies revealed that the change in GM volume of stroke patients was associated with neurological deficits (Lukic et al. 2017). However, it has not been established that QSM and GM volume could be used to predict the stroke severity of patients with SAO. The primary goal of this study was to explore the potential predictive value of QSM and GM volume in patients with SAO, which was expected to supply new clues for us to deeply understand SAO.

## 2 Materials and Methods

### 2.1 Participants

During the period of April to November 2023, a total of 66 patients (41 males and 25 females, age 68 [IQR 59.8–74] years) with SAO who met the inclusion criteria were enrolled in this study. All the participants provided written informed consent. Patients who met the following criteria were consecutively recruited: (1) age ≥ 18 years old; (2) acute ischemic stroke confirmed by MR imaging (MRI) with SAO according to the TOAST classification; (3) cranial MRI examination completed within 24 h of first onset. Patients (1) who received intravenous thrombolysis and/or endovascular therapy, (2) who had a history of major neurologic or psychiatric diseases or other clinically significant diseases, (3) who suffered from intracranial tumors and hemorrhagic stroke, and (4) who were unable to cooperate or understand the study procedures were excluded.

### 2.2 Clinical Information

For all of the included patients, the following basic clinical information was collected: age, sex, history of atrial fibrillation (AF), hypertension, diabetes mellitus, smoking, NIHSS score at admission, and laboratory results, such as total cholesterol (TC), triglycerides (TG), low‐density lipoprotein (LDL), high‐density lipoprotein (HDL), homocysteine (HCY), fibrinogen (Fbg), and d‐dimer.

According to the NIHSS score, patients were divided into two different groups: SAO patients with mild stroke (NIHSS scores < 4, 41 patients, abbreviated as SAO‐MiS) and SAO patients with moderate stroke (NIHSS scores ≥ 4, 25 patients, abbreviated as SAO‐MoS).

### 2.3 Image Acquisition

All participants underwent MRI examinations using a 3.0 T MR scanner (Siemens, Germany). T1‐weighted images were acquired with a 3D fast spoiled gradient‐echo sequence, and the parameters were as follows: TR =  1900 ms, TE = 2.01 ms, flip angle = 9°, field of view (FOV) = 256 mm × 256 mm, voxel size = 1 × 1 × 1 mm^3^, 176 slices, slice thickness = 1 mm.

The SWI sequence was acquired using a multi‐echo 3D GRE with the following parameters: TR = 50 ms, first TE = 6.84 ms, last TE = 43.74 ms, number of echoes = 10, bandwidth = 350 kHz, flip angle = 15°, field of view =  195 mm × 256 mm, voxel size = 0.75 × 0.75 × 2 mm^3^.

### 2.4 Infarct Region of Interest (ROI) Drawing

**FIGURE 1:**
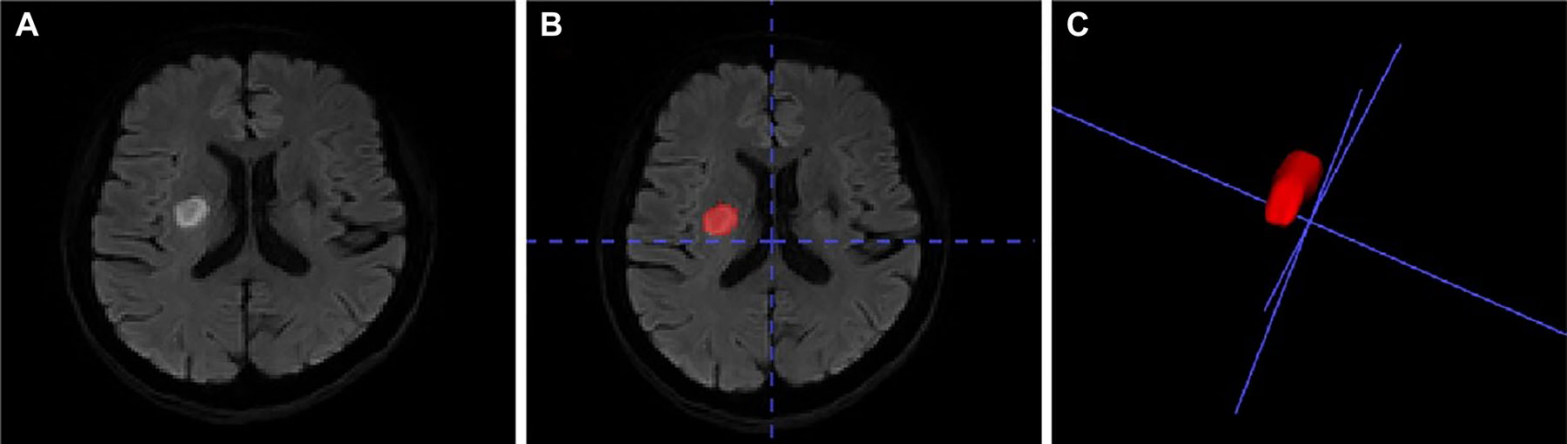
Case illustration of drawing infarct region of interest (ROI). Magnetic resonance (MR) images of a stroke patient aged 55 years with SAO. The DWI (A) shows the hyperintense infarct area on the right hemisphere. ROI (B) was drawn on the DWI image as depicted by the red contour using the ITK‐SNAP software. Then all ROIs on the total infarct slices of the patient were merged to an irregular red object (C).

First, two radiologists who were blinded to the clinical data reviewed the MR images to confirm the diagnosis and lesion position. Second, the DWI images were opened with the ITK‐SNAP software (http://www.itksnap.org/pmwiki/pmwiki.php), and the ROIs of lesions were manually drawn on the corresponding hyperintense regions on the DWI images of all the infarct slices for each patient, as illustrated in Figure 1. Third, total ROIs on all infarct slices of each patient were merged to an irregular red object, which was named with 3D “infarct ROI.”

### 2.5 QSM Mapping Preprocessing

We used the standard pipeline in the SEPIA (SuscEptibility mapping PIpeline tool for phAse images) toolbox in MATLAB (MathWorks, Natick, MA) to generate the QSM maps from GRE data (Chan and Marques 2021). We used the BET tool in FMRIB Software (FSL) from the MEDI toolbox to perform brain extraction on whole‐brain magnitude data. In short, (1) total field recovery: The optimum weights method was used to perform echo phase combination (Robinson et al. 2017) and SEGUE was used to perform phase unwrapping (Karsa and Shmueli 2019). (2) Background field removal: After unwrapping, the background field was removed using the Laplacian boundary value (LBV) (Zhou et al. 2014). (3) QSM dipole inversion: Magnetic susceptibility was quantitatively calculated using the STAR‐QSM method, and QSM maps were generated (Wei et al. 2017).

### 2.6 Voxel‐Based Morphometry Analysis

Voxel‐based morphometry (VBM) analysis was performed using the DPABI software, built on the MATLAB R2020b (MathWorks, Natick, MA, USA). Initially, 3D T1‐weighted images were segmented into GM, white matter (WM), and cerebrospinal fluid (CSF) utilizing the diffeomorphic anatomical registration through exponentiated lie algebra (DARTEL) algorithm. Subsequently, the GM segments were spatially normalized to the Montreal Neurological Institute (MNI) standard space using DARTEL registrations. Following normalization, the GM images were modulated to correct for volume changes incurred during the normalization process. This modulation step involved multiplying the voxel values by the Jacobian determinants derived from the spatial normalization step. Finally, the modulated and normalized images were smoothed using an 8 mm full width at half maximum (FWHM) Gaussian kernel (Nemoto 2017; Chen, Chen et al. 2022).

### 2.7 QSM Value and GM Volume Within the ROI

For each subject, the mean quantitative susceptibility and GM volume within the infarct ROI were calculated using in‐house code in MATLAB.

### 2.8 Statistical Analyses

#### 2.8.1 Demographics and Clinical Characteristics

Two‐sample *t*‐tests and chi‐square tests were used to compare differences in demographic data and clinical characteristics between SAO‐MiS and SAO‐MoS (*p* < 0.05).

#### 2.8.2 QSM Value and GM Volume of Infarct ROI in SAO‐MiS and SAO‐MoS

Two‐sample *t*‐tests were used to compare differences in QSM value and GM volume of the infarct ROI between SAO‐MiS and SAO‐MoS (*p* < 0.05).

#### 2.8.3 Comparisons of Voxel‐Wise GM Volume on Whole‐Brain Voxel Level Between SAO‐MiS and SAO‐MoS

First, the GM images were registered to the AAL3v1 atlas and resampled to a voxel size of 2 mm × 2 mm × 2 mm. Voxel‐wise comparisons of GM volume between SAO‐MiS and SAO‐MoS were conducted within GM masks, controlling for age and sex (with a TFCE‐corrected *p* < 0.05 and a minimum cluster size of > 240 mm^3^).

#### 2.8.4 Relationship Among QSM Value, GM Volume of Infarct ROI, Voxel‐Wise GM Volume, and Clinical Variables

We extracted the QSM values, GM volumes of infarct ROIs, and voxel‐wise GM volumes from brain regions that exhibited significant voxel‐wise GM volume differences between SAO‐MiS and SAO‐MoS. Subsequently, correlation analyses between clinical variables and QSM values and GM volumes of infarct ROI were performed separately for all enrolled patients by partial correlation analyses.

#### 2.8.5 Diagnostic Efficacy of the Combination of QSM Value, GM Volume of Infarct ROI, and Voxel‐Wise GM Volume

A support vector machine (SVM) approach was applied to distinguish SAO‐MoS from SAO‐MiS subjects. A leave‐one‐out cross‐validation (LOOCV) strategy was used to assess the generalizability of this SVM classifier and to measure its sensitivity and specificity. Receiver‐operating characteristic (ROC) curves were applied to evaluate the diagnostic efficacy of the combination of the QSM value, GM volume of infarct ROI, and voxel‐wise GM volume, with the area under the curve (AUC) being considered diagnostically valuable when AUC > 0.7.

## 3 Results

### 3.1 Demographic and Clinical Characteristics

Sixty‐six patients with SAO were recruited in this study. According to the results of NIHSS scores, they were divided into SAO‐MiS and SAO‐MoS groups. Median NIHSS was 2.32 (IQR 2–3) for the SAO‐MiS group, whereas the median NIHSS was 5.12 (IQR 4–5.5) for the SAO‐MoS group, and the maximum NIHSS in the moderate category is 11. There were no significant differences in terms of age, sex, and all clinical characteristics between SAO‐MiS and SAO‐MoS (*p* > 0.05). The detailed clinical characteristics of all subjects are summarized in Table 1.

**TABLE 1:** Demographic and clinical characteristics of all participants.

Items	SAO‐MiS (*n* = 41)	SAO‐MoS (*n* = 25)	*p* value
Age, M (IQR)	67.0 (55.5–75.5)	68.0 (63.0–73.5)	0.386
Gender, male(%)	25 (61.0)	19 (76.0)	0.209
AF, *n* (%)	2 (4.9)	0 (0)	0.522
Hypertensive, *n* (%)	23 (56.1)	18 (72.0)	0.196
Diabetes, *n* (%)	13 (31.7)	9 (36.0)	0.720
Smoker, *n* (%)	17 (41.5)	15 (60.0)	0.236
TG, M (IQR)	1.31 (1.10–2.14)	1.50 (0.90–1.82)	0.924
TC, mean ± SD	4.54 (0.87)	4.26 (1.10)	0.265
LDL‐cholesterol, mean ± SD	2.98 (0.87)	2.71 (0.90)	0.243
HDL‐cholesterol, mean ± SD	0.93 (0.18)	0.91 (0.21)	0.667
Fibrinogen, mean ± SD	3.15 (0.65)	3.06 (0.57)	0.611
d‐Dimer, M (IQR)	0.39 (0.19–0.54)	0.41 (0.28–0.68)	0.402
Homocysteine, M (IQR)	13.2 (12.1–21.4)	15.1 (13.1–19.5)	0.427

Abbreviations: AF, atrial fibrillation; HDL, high‐density lipoprotein; LDL, low‐density lipoprotein; SAO‐MiS, mild stroke with small artery occlusion; SAO‐MoS, moderate stroke with small artery occlusion; TC, total cholesterol; TG, triglyceride.

### 3.2 Differences in QSM Value, GM Volume of Infarct ROI, and Voxel‐Wise GM Volume Between SAO‐MiS and SAO‐MoS

**FIGURE 2:**
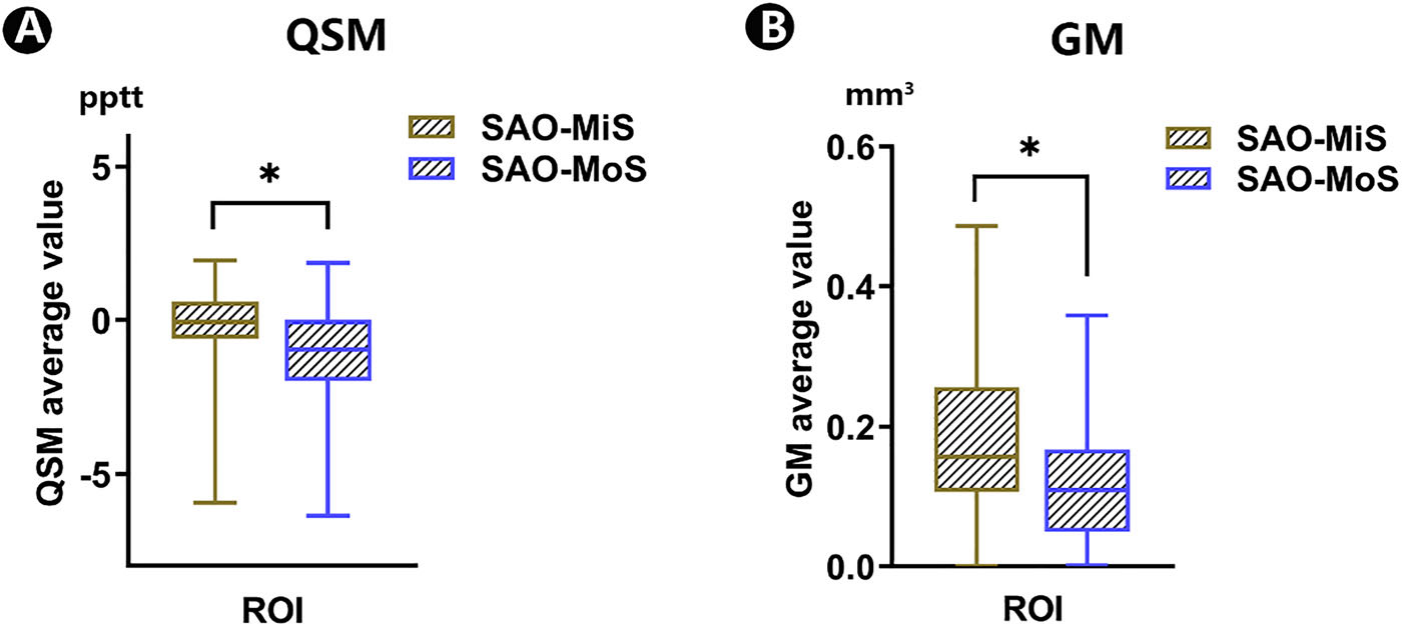
Differences in QSM value and GM volume of infarct ROI between SAO‐MiS and SAO‐MoS. GM, gray matter; QSM, quantitative susceptibility mapping; ROI, region of interest; SAO‐MiS, mild stroke with small artery occlusion; SAO‐MoS, moderate stroke with small artery occlusion.

**FIGURE 3:**
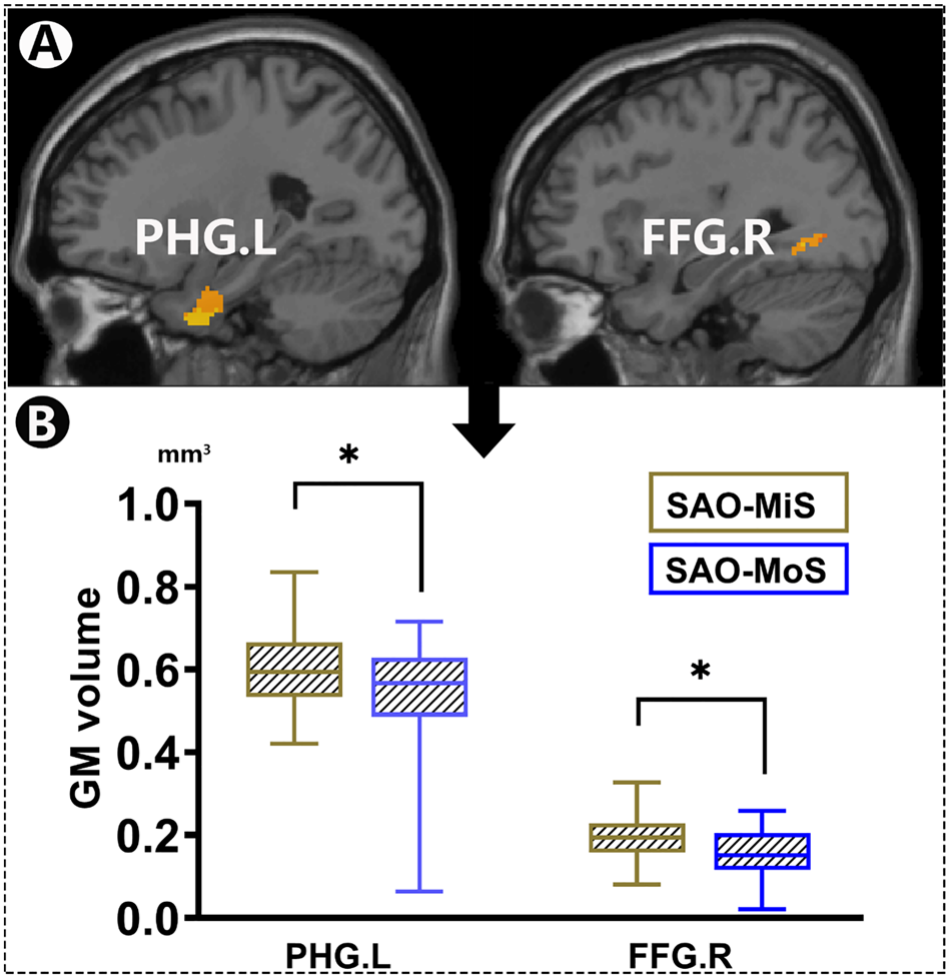
Differences in voxel‐wise GM volume measured using VBM between SAO‐MiS and SAO‐MoS. FFG.R, right fusiform gyrus; GM, gray matter; L, left hemisphere; PHG.L, left parahippocampal gyrus; R, right hemisphere; SAO‐MiS, mild stroke with small artery occlusion; SAO‐MoS, moderate stroke with small artery occlusion.

As shown in Figure 2, compared with SAO‐MiS, SAO‐MoS had lower QSM values and lower GM volume of the infarct ROI. In addition, as depicted in Figure 3, SAO‐MoS exhibited a lower GM volume in the left parahippocampal gyrus (PHG.L) and right fusiform gyrus (FFG.R) compared with SAO‐MiS on the whole brain level.

### 3.3 Relationship Among QSM Value, GM Volume of Infarct ROI, Voxel‐Wise GM Volume, and Clinical Variables

**FIGURE 4:**
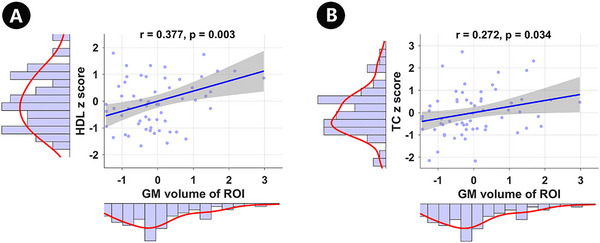
Relationship (A and B) between GM volume of infarct ROI and clinical variables. GM, gray matter; HDL, high‐density lipoprotein; ROI, region of interest; TC, total cholesterol.

As indicated in Figure 4, the GM volume of the infarct ROI was positively correlated with both HDL (*r* = 0.377, *p* = 0.003) and TC levels (*r* = 0.272, *p* = 0.034). Besides, we also analyzed the correlations between GM volume in the left PHG and GM volume in the right FFG with the clinical variables (such as Fbg, HCY, LDL, HDL, TC, TG, and d‐dimer), respectively, but most of the results did not show the significant differences. Those negative results are reported as Data S1.

### 3.4 Distinguishing SAO‐MoS From SAO‐MiS Subjects Using a Combination of QSM Value, GM Volume of Infarct ROI, and Voxel‐Wise GM Volume

**FIGURE 5:**
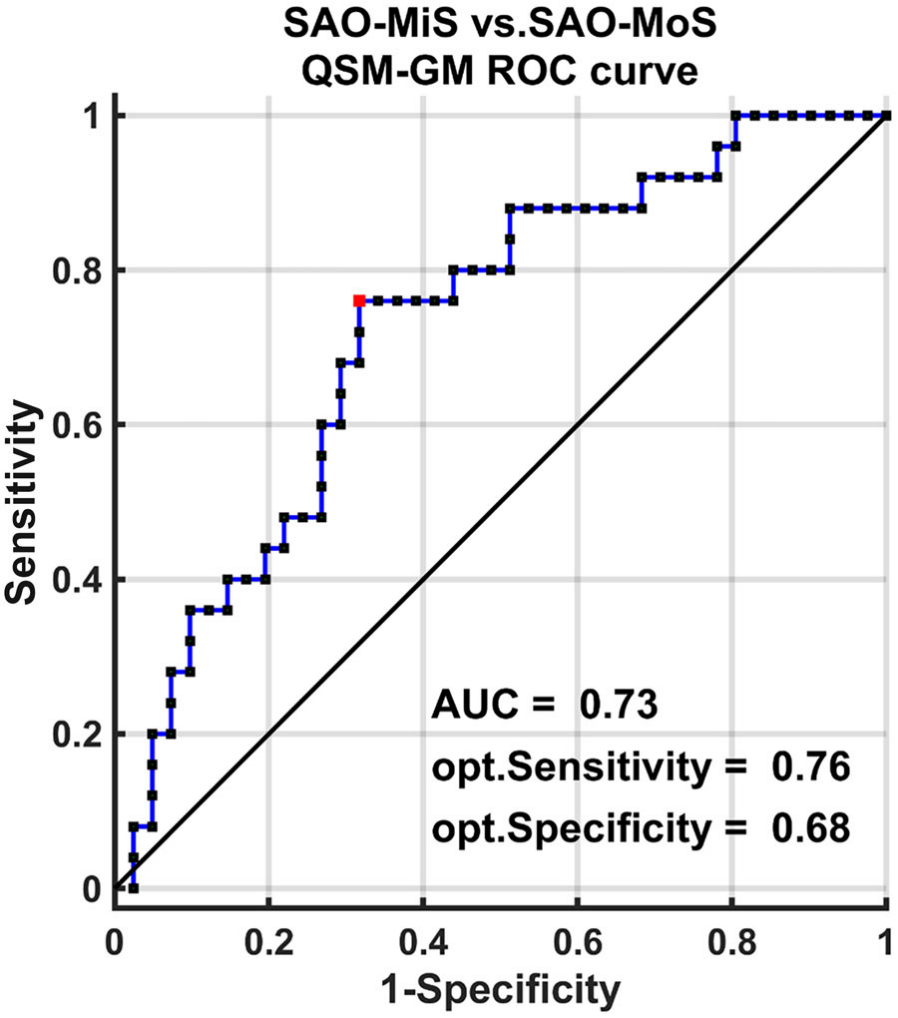
Classification of individuals as SAO‐MiS and SAO‐MoS using a combination of QSM value, GM volume of infarct ROI, and voxel‐wise GM volume. ROC curve shows the classification power in the “he ve shows theSAO‐MoS from SAO‐MiS. AUC, the area under the ROC curve; Opt, optimum; ROC, receiver‐operating characteristic; SAO‐MiS, mild stroke with small artery occlusion; SAO‐MoS, moderate stroke with small artery occlusion.

SAO‐MoS showed significantly different QSM values, GM volumes of the infarct ROI, and voxel‐wise GM volumes in the PHG.L and FFG.R compared to SAO‐MiS (Table 2, Figures 2 and 3). This finding led us to further explore the potential of using these significant different factors to distinguish patients with SAO‐MiS from those with SAO‐MoS. However, the results showed the predictive efficiency is not very high if we only use the lesion GM volume or QSM value to distinguish SAO‐MiS from SAO‐MoS. Those relevant negative results have been reported as Data S2. According to the recent consensus that multi‐modality MRI plays a more accurate role in the diagnosis of many diseases than traditional single‐modality MRI. We try to explore the predictive efficiency of a combination of QSM values, GM volumes of the infarct ROI, and voxel‐wise GM volumes to distinguish patients with SAO‐MiS from those with SAO‐MoS. As shown in Figure 5, the SVM classifier's ROC curve displayed a strong ability of this model to differentiate SAO‐MoS from SAO‐MiS on an individual subject basis, with an AUC of 73%, sensitivity of 76%, and specificity of 68%.

**TABLE 2:** Comparisons of gray matter volume measured using voxel‐based morphometry (VBM) between mild stroke with small artery occlusion (SAO‐MiS) and moderate stroke with small artery occlusion (SAO‐MoS) subjects.

		MNI				
Brain regions	L/R	*x*	*y*	*z*	*T* values	Cluster size (mm^3^)
**SAO‐MiS > SAO‐MoS**						
Parahippocampal gyrus	L	− 24	2	− 42	3.2193	1424
Fusiform gyrus	R	32	− 60	− 6	2.667	296

*Note*: Regions with significant voxel numbers ≥ 30 with TFCE correction were presented and defined from the regions of interest (ROIs) in the AAL3v1 atlas.

Abbreviations: L, left hemisphere; MNI, Montreal neurological institute; R, right hemisphere.

## 4 Discussion

To our knowledge, this is the first study to assess the severity of neurological deficits by integrating multiple MRI parameters in SAO patients. Using MRI data from the acute SAO patients, we demonstrated that both the mean value of QSM and GM volumes of infarct ROI in SAO‐MoS group were lower than SAO‐MiS group. Moreover, SAO‐MoS exhibited lower GM volumes in the PHG.L and FFG.R compared with SAO‐MiS group. Specifically, the combination of decreased QSM value and GM volume of infarct ROI and voxel‐wise GM volumes was a strong predictor of neurological deficit in SAO patients.

Our study first demonstrated that the mean QSM value and GM volume within the infarct ROI in SAO‐MoS group were lower than SAO‐MiS group. QSM has been mainly reported as useful to evaluate vessel function and oxygen metabolism in ischemic stroke patients (Haacke et al. 2010; Belachew et al. 2021; Chen, Zhang et al. 2022c). A previous study found the increased susceptibility of asymmetrically prominent cortical veins (APCVs) correlated with misery perfusion and poor outcomes in patients with occlusion of the middle cerebral artery (Luo et al. 2017). However, Probst et al. (2021) found no correlation of venous susceptibility values within the infarct area after successful recanalization with infarct size or outcome. It seems the predictive function of venous susceptibility in patients with ischemic stroke is still unclear. Some researchers have started to focus on the relationship between magnetic susceptibility of thrombus and clinical outcome of stroke patients. A study by Chen, Zhang et al. (2022) showed that magnetic susceptibility in basilar artery thrombus could discriminate cardiogenic embolism from other stroke subtypes. Chen, Zhang et al. (2022b) also found that increased thrombus magnetic susceptibility was associated with successful recanalization and favorable clinical outcomes for stroke patients with middle cerebral artery occlusion. Our study distinctively paid attention to the magnetic susceptibility of infarct area in patients with SAO. Normally, Paramagnetic materials have positive susceptibility, whereas diamagnetic materials have negative susceptibility. The magnetic susceptibility of water in the brain is about –9.035 ppm, whereas the variations of susceptibility among brain tissues relative to water are about ± 0.1 ppm (Liu et al. 2015). The GM mainly appears paramagnetic and the WM largely diamagnetic in a healthy adult brain. The potential consensus is that the GM's paramagnetic susceptibility is mostly related to iron and the WM's diamagnetic susceptibility is mainly due to myelination (Shmueli et al. 2009; Li, Wu, and Liu 2011). Additionally, copper deposition, calcification, the concentration of paramagnetic deoxyhemoglobin, and diamagnetic oxyhemoglobin are other common sources that affect susceptibility in the brain (Pauling and Coryell 1936; Schweser et al. 2010; Skowrońska et al. 2013). To our knowledge, the magnetic susceptibility characteristics of brain infarct core are still markedly less clear. A study in mice found higher susceptibility values in the ischemic hemisphere after transient middle cerebral artery occlusion (Vaas et al. 2018). In our study, we found two magnetic susceptibility related features within the infarct area: One is that the QSM values of infarct areas of both the SAO‐MiS and SAO‐MoS groups are negative; another one is that SAO‐MoS had lower QSM values than the SAO‐MiS, which seems in contrast to the previous mice model QSM study. One supposed reason is that the dead brain tissues already have lost the ability to utilize the oxygen, which eventually increased the partial concentration of diamagnetic oxyhemoglobin. Certainly, as mentioned above, it is very difficult to specifically interpret the susceptibility values of brain tissues since the effects of multiple signal sources. Our study first revealed the susceptibility characteristics of acute infarct brain tissues. Further studies are needed to investigate magnetic changes after acute ischemic stroke lesions.

Besides, we also found SAO‐MoS had lower GM volume within the infarct ROI compared to SAO‐MiS. The changes of QSM value were proved to be related to the decreases of GM volume, which is also proved to be associated with neurological deficits (Chai et al. 2015). In consideration of the relationship between QSM and GM volume, we furtherly detect the GM volume within the ischemic core of the two groups by QSM. GM volume changes are related to many chronic brain vascular disorders. Lukic et al. (2017) reported that increased GM volume in the middle temporal gyrus (MTG) and the supplementary motor area (SMA) of the right hemisphere was associated with better language comprehension and production scores. Another study demonstrated that the GM volume increase in the SMA may facilitate motor recovery in subcortical stroke patients (Diao et al. 2017). A recent study found the GM volume of middle frontal gyrus (MFG) together with the clinical and demographic variables could be concerned as a predictive model to distinguish post‐stroke depression (PSD) from the non‐PSD patients (Hong et al. 2020). However, the relationship of GM volume and acute ischemic stroke severity has not been explored. The present study showed that, compared with SAO‐MiS, SAO‐MoS had a lower GM volume of the infarct ROI. Additionally, by adopting the voxel‐wise GM volume on the whole brain for each participant, we also fund some significant difference of the GM volume distribution in some specifical brain regions between the two groups. On whole‐brain voxel level, we found SAO‐MoS exhibited a lower voxel‐wise GM volume in the PHG.L and FFG.R compared with SAO‐MiS. Our study is one of the first studies to explore the correlation of GM volumetric changes in the infarct area and the whole brain with neurological deficits in patients with SAO. Our finding is that SAO‐MoS had lower GM volume within infarct core; the underlying reason may be that higher NIHSS scores are usually related to more serious injuries of brain neurons, which eventually leads to the decrease of GM volume. However, how to explain the lower volumes in PHG.L and FFG.R of the SAO‐MoS group still needs our further study.

Subsequently, to explore the underlying mechanism of our finding, we analyzed the relationship among QSM value, GM volume of infarct ROI, and various clinical variables. The result showed only the GM volume of the infarct ROI was positively correlated with both HDL and TC levels. Previous studies indicated that HDL may be associated with altered regional brain volumetric data in Alzheimer's disease (Pedrini et al. 2022). Another study dedicated to low HDL was associated with low hippocampal volume in individuals with mild cognitive impairment and healthy older controls, which was consent to our results in a way (Wolf et al. 2004). Cholesterol is a vital structural component of myelin and essential for brain homeostasis. The present fact is that high TC level has been proved to be a strong predictor of cardiovascular disease (Peters et al. 2016), whereas the relationship between cholesterol and stroke has been inconsistent. Moreover, the relevance of cholesterol level and GM volume is still undefined. A study showed higher TC levels were linked to increased GM volume in the frontal cortex in hypertensive adults (Chung et al. 2018). Yang, Stanford, and Jiang (2020) reported low cholesterol levels linked to reduced GM volume in the medial temporal lobe in Parkinson's disease patients. The molecular mechanism by which the plasma lipid metabolism affects GM volume needs to be investigated with further studies.

NIHSS is a well‐known scale to evaluate the degree of neurological deficits and final outcomes in acute ischemic stroke (König et al. 2008; Saver and Altman 2012). However, NIHSS exhibits certain limitations. First, the NIHSS score might be constrained in accurately evaluating posterior circulation strokes as it was primarily designed to examine anterior circulation strokes. Second, NIHSS tends to pay more attention to dysfunction associated with left hemisphere lesions than with right hemisphere lesions. Additionally, NIHSS is a subjective assessment, which means the results are usually affected by many inevitable artificial errors. This study tries to find another reliable method to assess the severity of SAO patients’ neurological deficits. It is a pity that few studies have researched the features of QSM value and GM volume in patients with SAO and their relationship with neurological deficits. Our study demonstrated that the combination of mean QSM value, GM volume in the infarct ROI, and voxel‐wise GM volumes could be applied to distinguish patients with SAO‐MiS from those with SAO‐MoS. Our findings will supply more new clinical clues for further understanding the SAO stroke. We have not found similar studies with our study. Confirmation and extension of our findings in larger research are needed.

This study has limitations. First, in the SVM part, only the two regions significantly different between the two groups were used to train the classifier, which may lead to an inadvertent “double‐dipping” problem. We will run the analysis using a completely random date by including more patients in the future work to solve this problem. Second, the present study is a single‐center study with a limited number of participants, and a large multi‐center cohort was required to validate our conclusion. Lastly, our study cannot explain whether neurological dysfunction in SAO patients leads to the changes of QSM value and GM volumes or whether the QSM value and GM volume's changes aggravate neurological deficits. To explore the true causal relationship between stroke deficit severity and the variation of QSM value and GM volumes, it is necessary to perform further prospective, longitudinal studies.

## 5 Conclusion

In conclusion, using QSM and T1 MRI information, we found SAO‐MoS patients had a lower QSM value and a lower GM volume of the infarct ROI than SAO‐MiS group. Additionally, SAO‐MoS exhibited a lower GM volume in the PHG.L and FFG.R compared with SAO‐MiS. Importantly, a combination of QSM values, GM volumes of the infarct ROI, and voxel‐wise GM volumes displayed a strong ability to differentiate SAO‐MoS from SAO‐MiS, which may provide us new insights to understand the neurological deficit in SAO stroke.

## Author Contributions

**Xuelian Tang**: software, validation, writing–original draft preparation, writing–review and editing. **Zhenzhen He**: formal analysis, data curation, writing–original draft preparation, visualization. **Qian Yang**: investigation. **Tao Yang**: supervision. **Yusheng Yu**: conceptualization, data curation, project administration. **Jinan Chen**: conceptualization, methodology, validation, resources, writing–review and editing, funding acquisition. All authors have read and agreed to the published version of the manuscript.

## Ethics Statement

This study was approved by the Medical Ethics Committee of Nanjing Jiangning Hospital (2023‐03‐074‐K01).

## Conflicts of Interest

The authors declare no conflicts of interest.

### Peer Review

The peer review history for this article is available at https://publons.com/publon/10.1002/brb3.70080.

## Supporting information



Supporting Information

Supporting Information

## Data Availability

The data that support the findings of this study are available on request from the corresponding author. The data are not publicly available due to privacy or ethical restrictions.
