# Supplementary figures and images for: Combining Quantitative Susceptibility Mapping With the Gray Matter Volume to Predict Neurological Deficits in Patients With Small Artery Occlusion
Source: Brain Behav. 2024 Oct 4;14(10):e70080. doi: 10.1002/brb3.70080 (PMC11450255; doi:10.1002/brb3.70080)

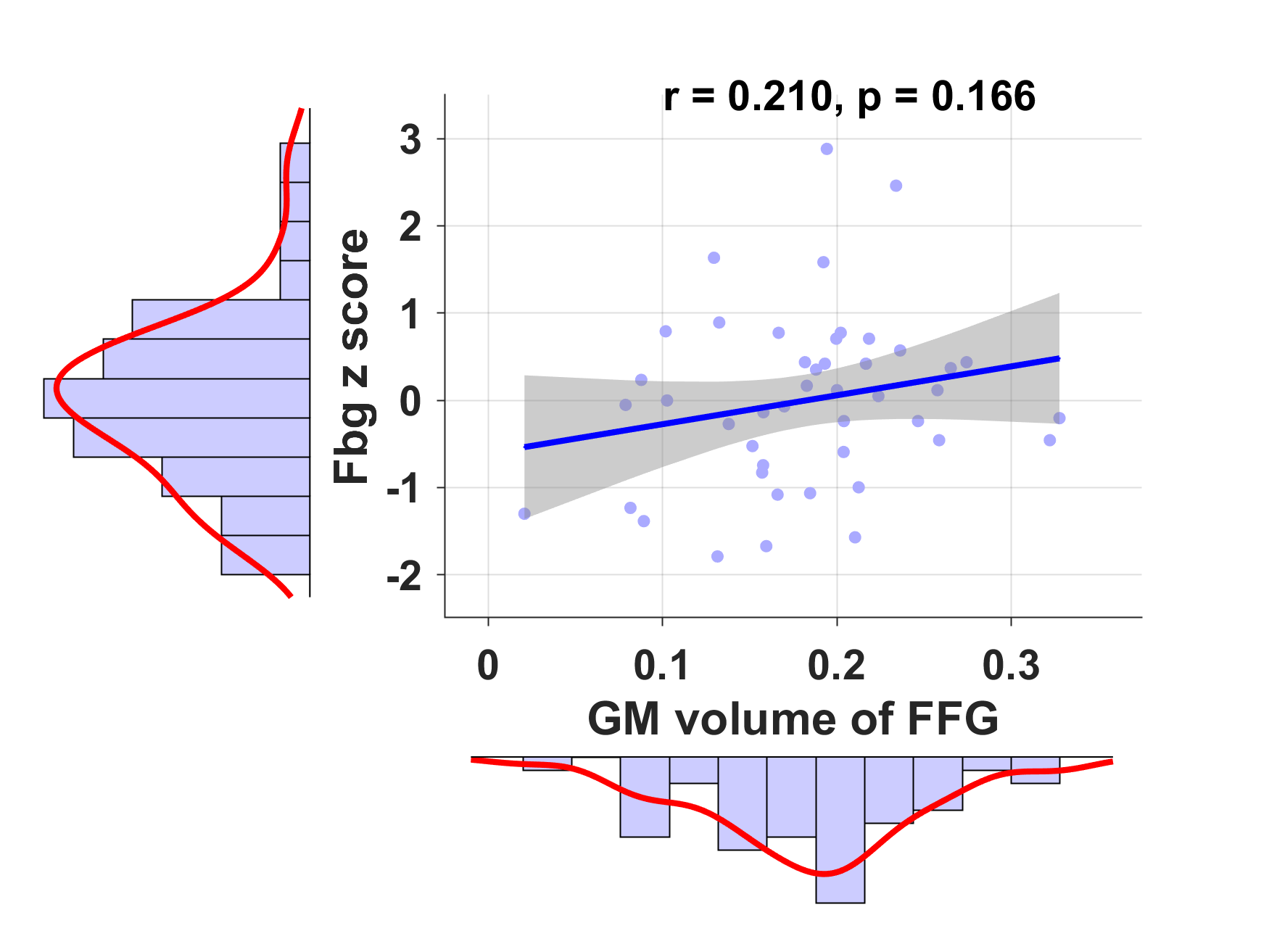

Supplement: Supplementary file 1 — Supporting Information [file BRB3-14-e70080-s002.zip › FFG GM-Fbg.tif]

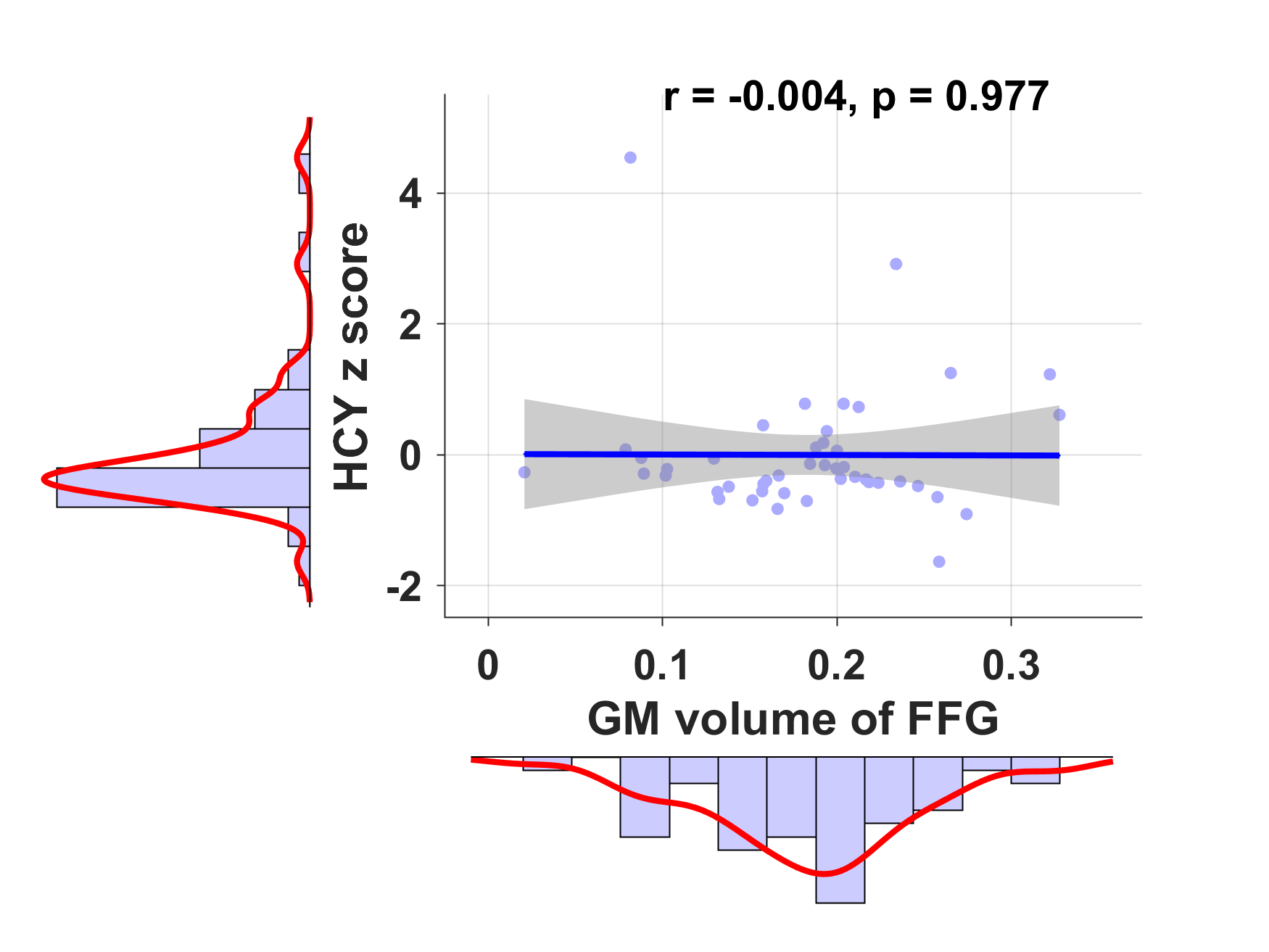

Supplement: Supplementary file 1 — Supporting Information [file BRB3-14-e70080-s002.zip › FFG GM-HCY.tif]

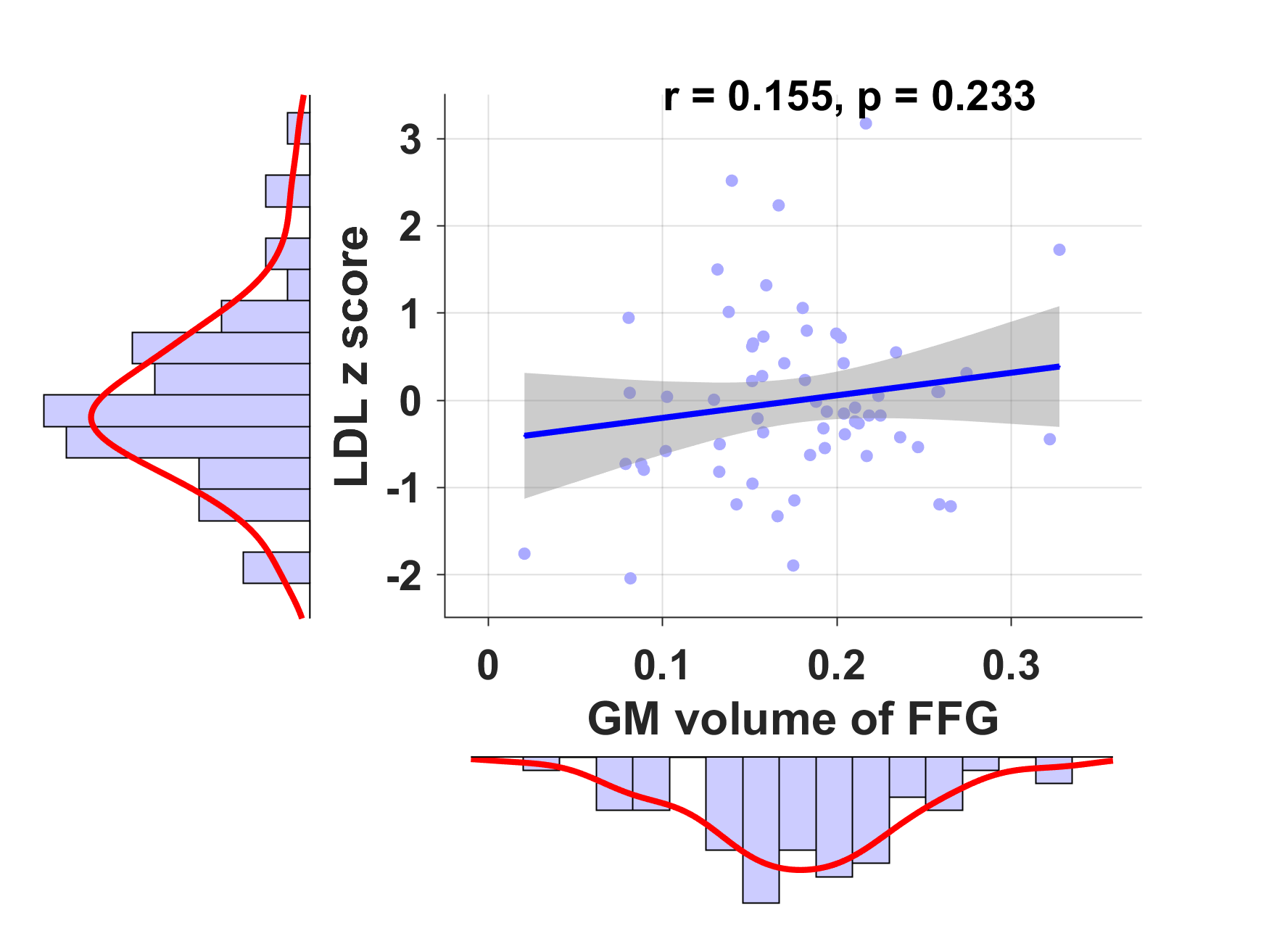

Supplement: Supplementary file 1 — Supporting Information [file BRB3-14-e70080-s002.zip › FFG GM-LDL.tif]

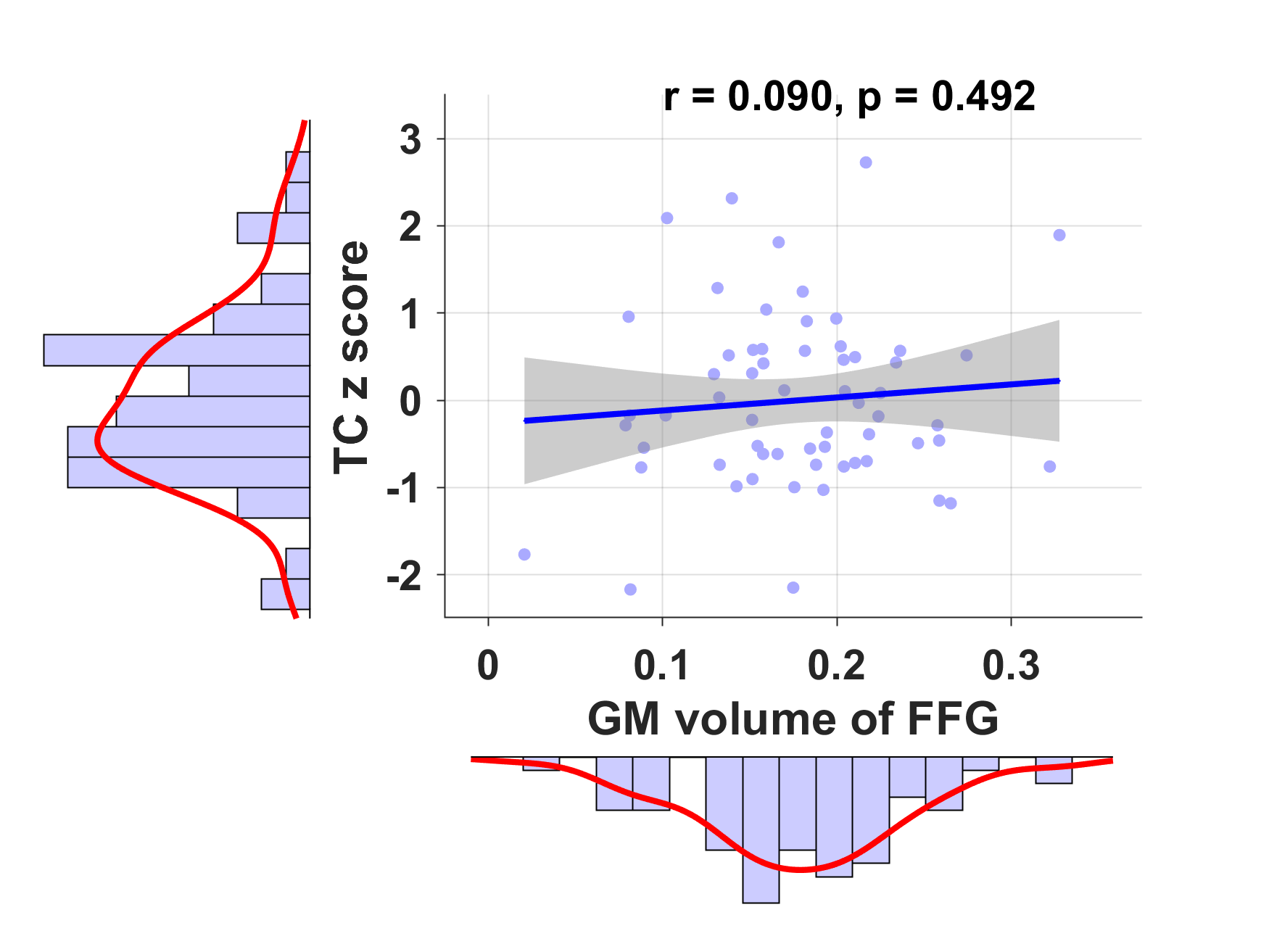

Supplement: Supplementary file 1 — Supporting Information [file BRB3-14-e70080-s002.zip › FFG GM-TC.tif]

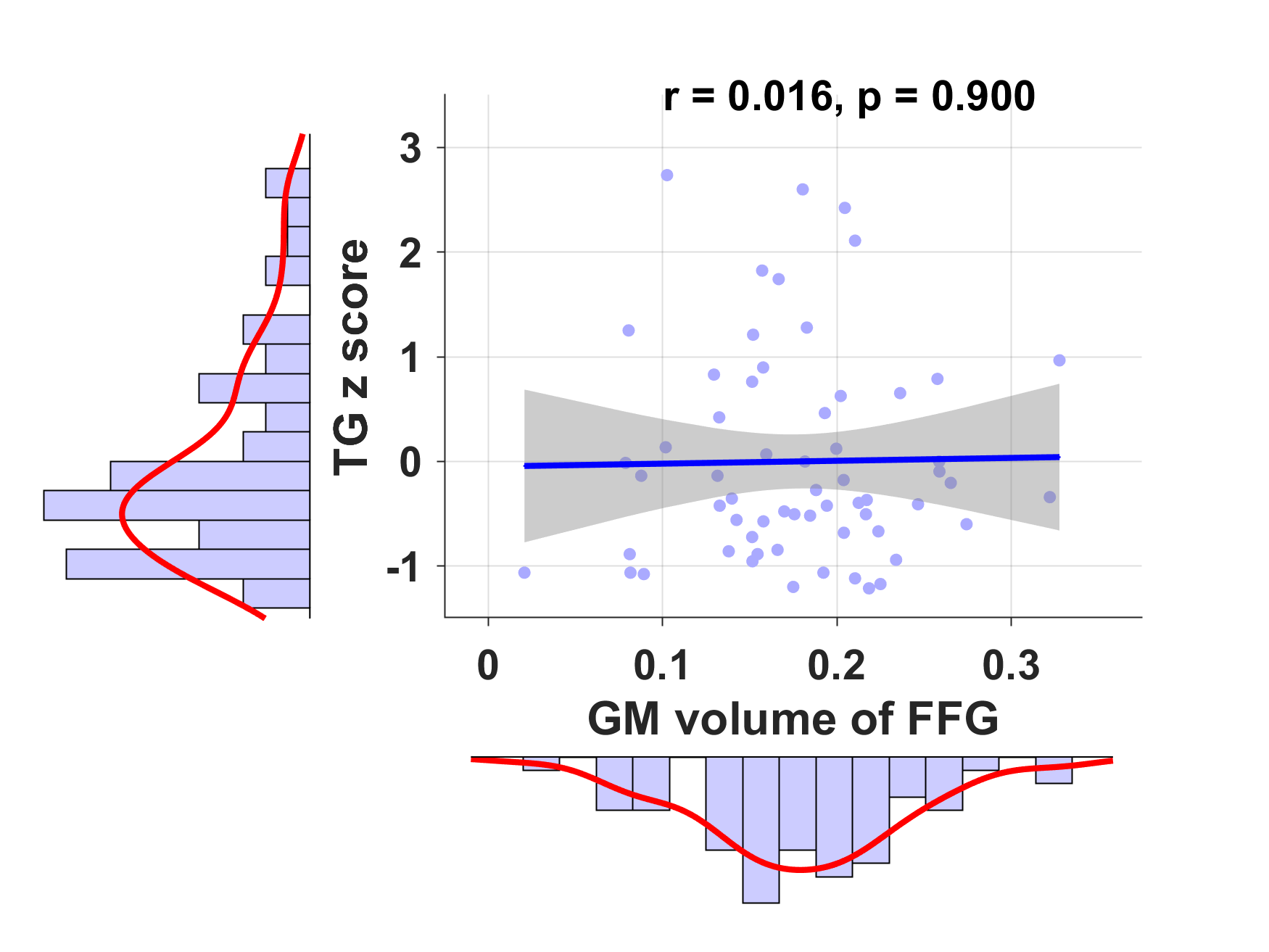

Supplement: Supplementary file 1 — Supporting Information [file BRB3-14-e70080-s002.zip › FFG GM-TG.tif]

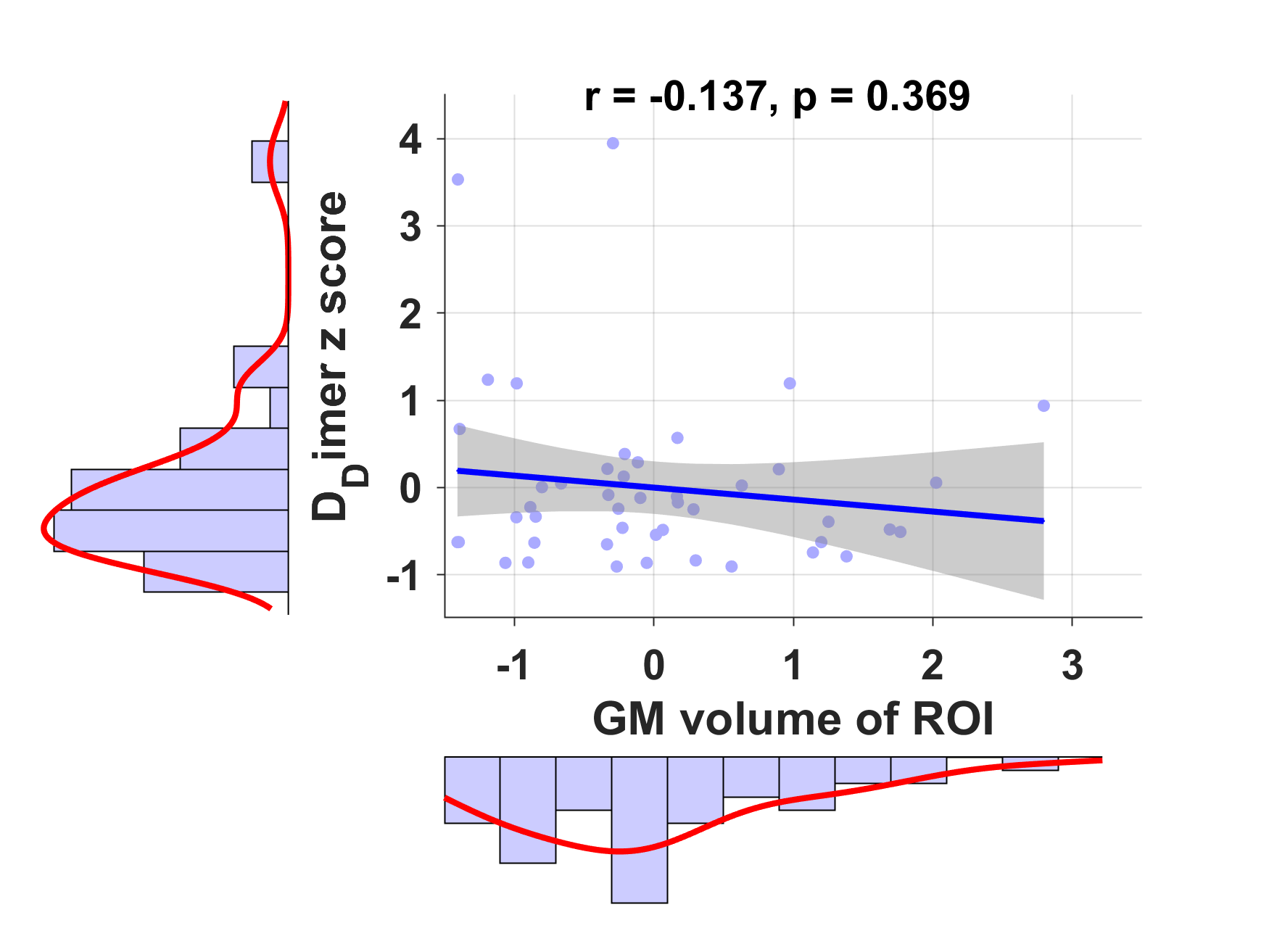

Supplement: Supplementary file 1 — Supporting Information [file BRB3-14-e70080-s002.zip › GMavg-D_Dimer.tif]

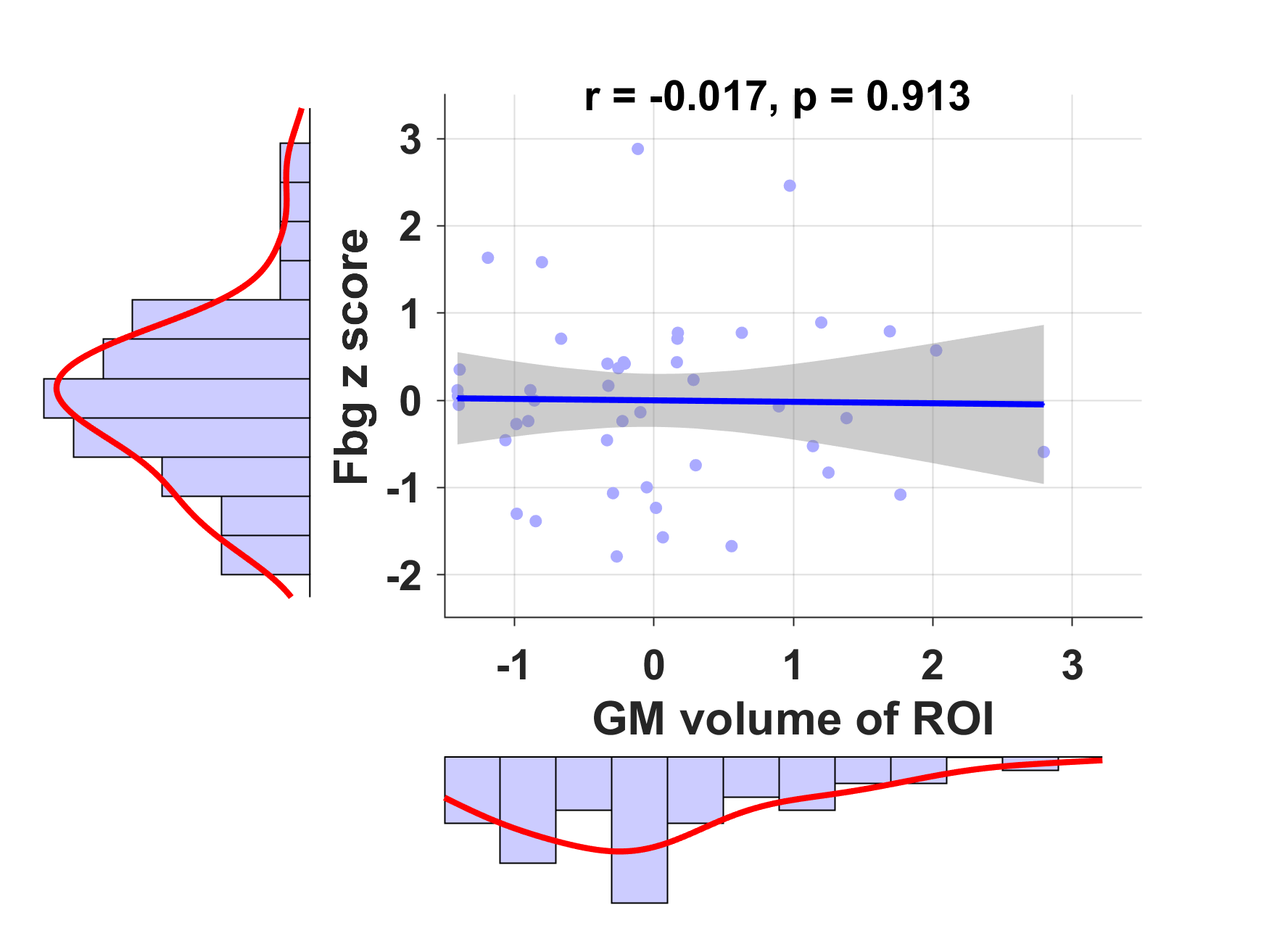

Supplement: Supplementary file 1 — Supporting Information [file BRB3-14-e70080-s002.zip › GMavg-Fbg.tif]

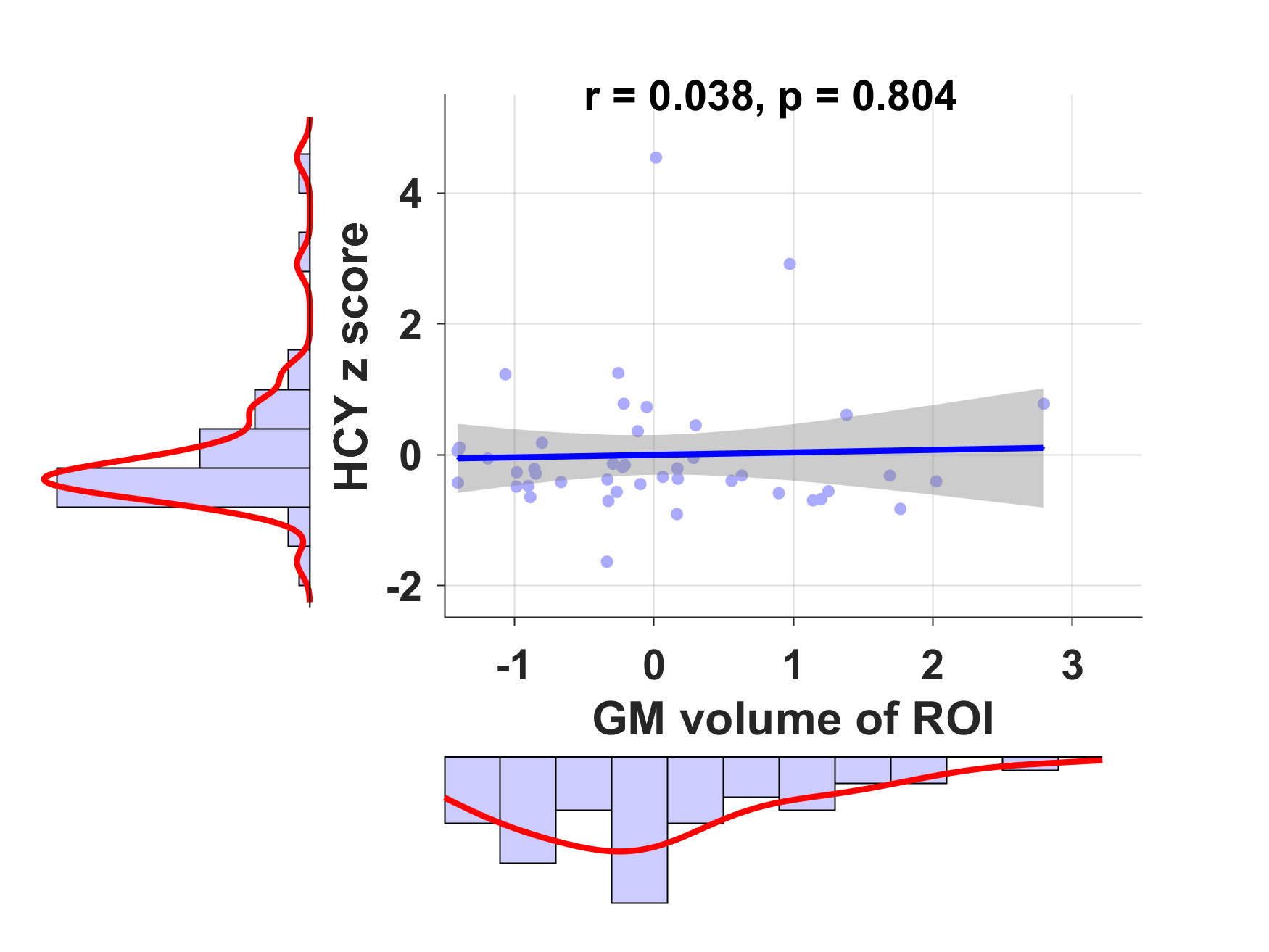

Supplement: Supplementary file 1 — Supporting Information [file BRB3-14-e70080-s002.zip › GMavg-HCY.tif]

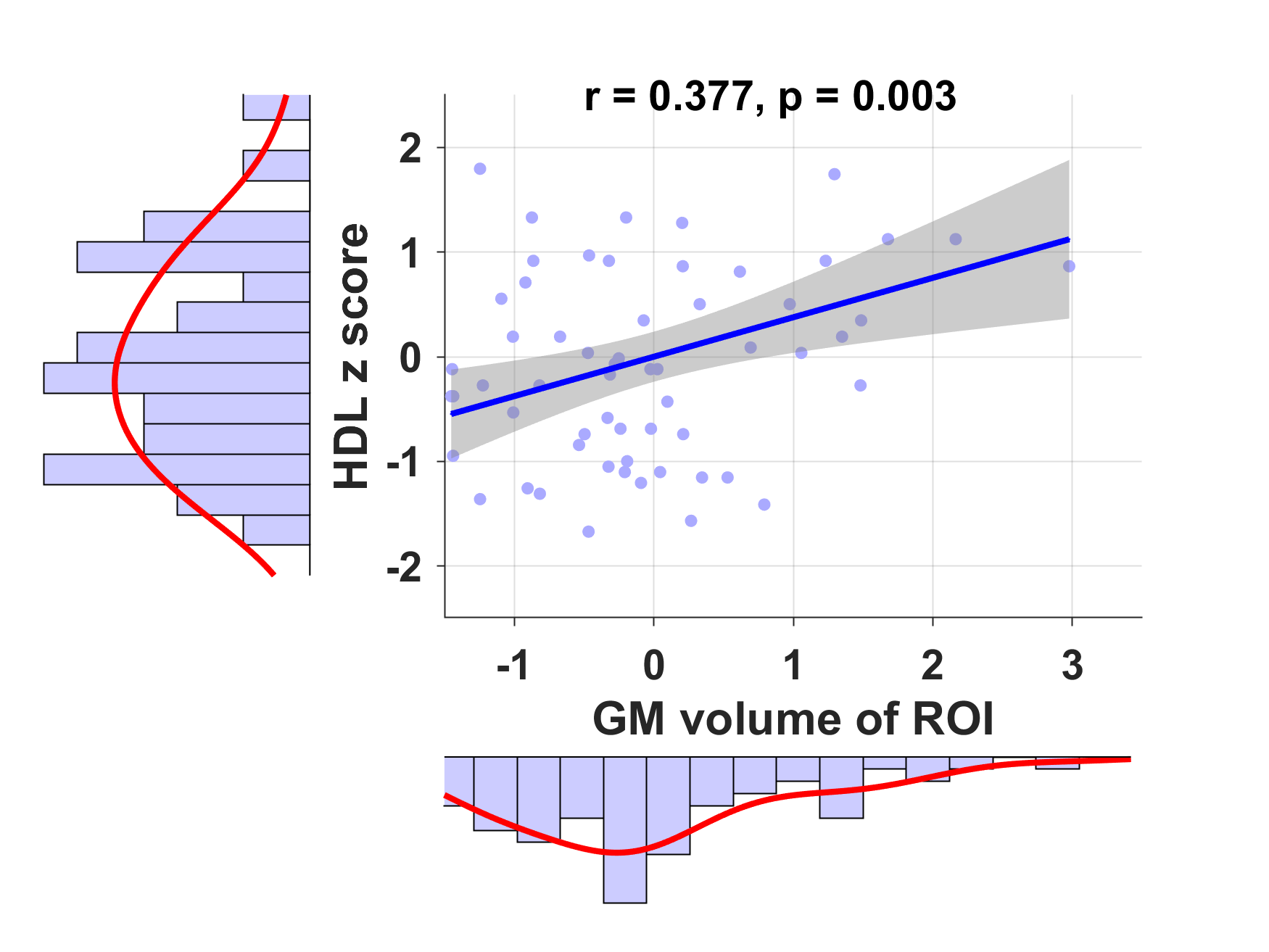

Supplement: Supplementary file 1 — Supporting Information [file BRB3-14-e70080-s002.zip › GMavg-HDL.tif]

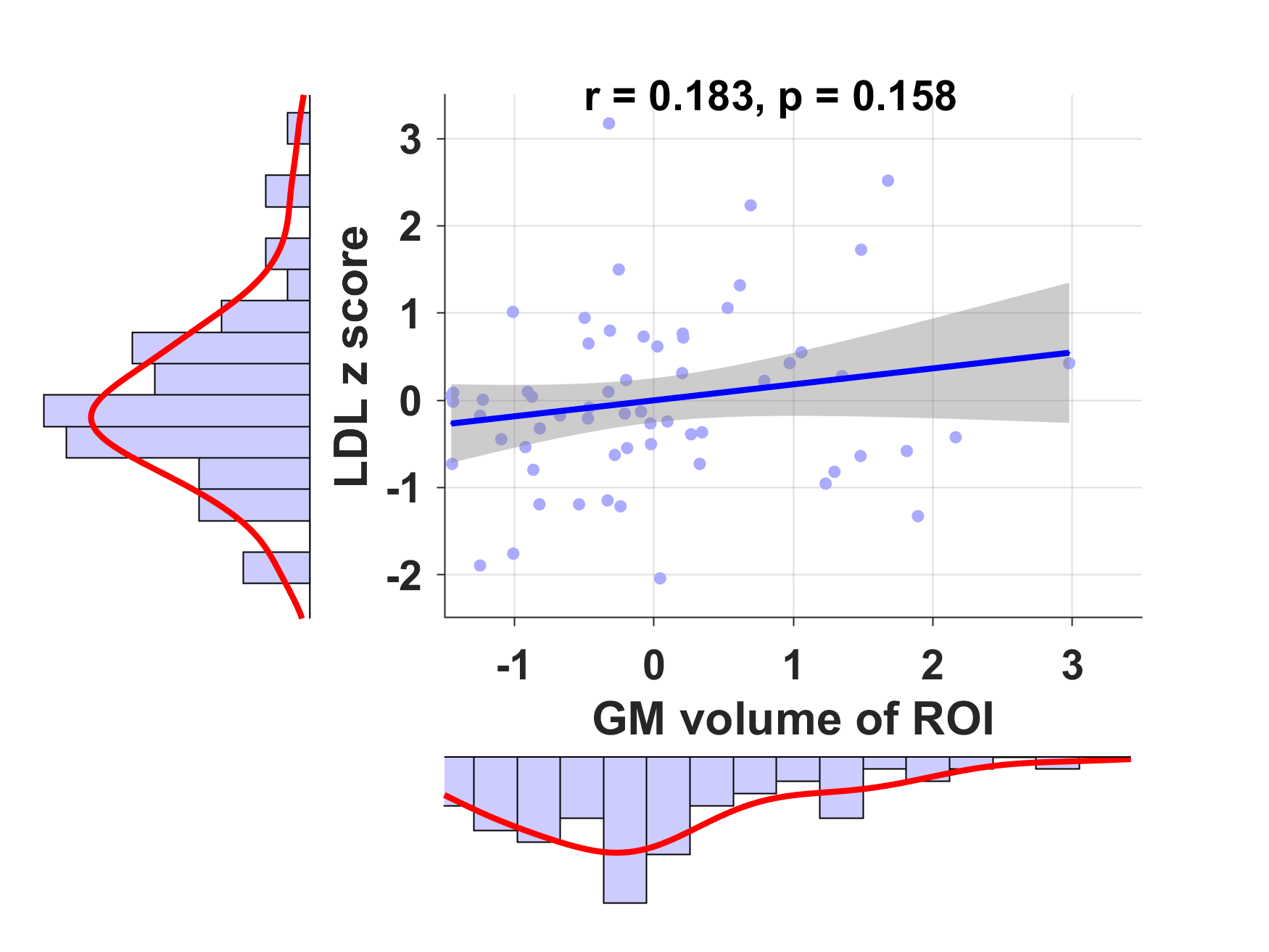

Supplement: Supplementary file 1 — Supporting Information [file BRB3-14-e70080-s002.zip › GMavg-LDL.tif]

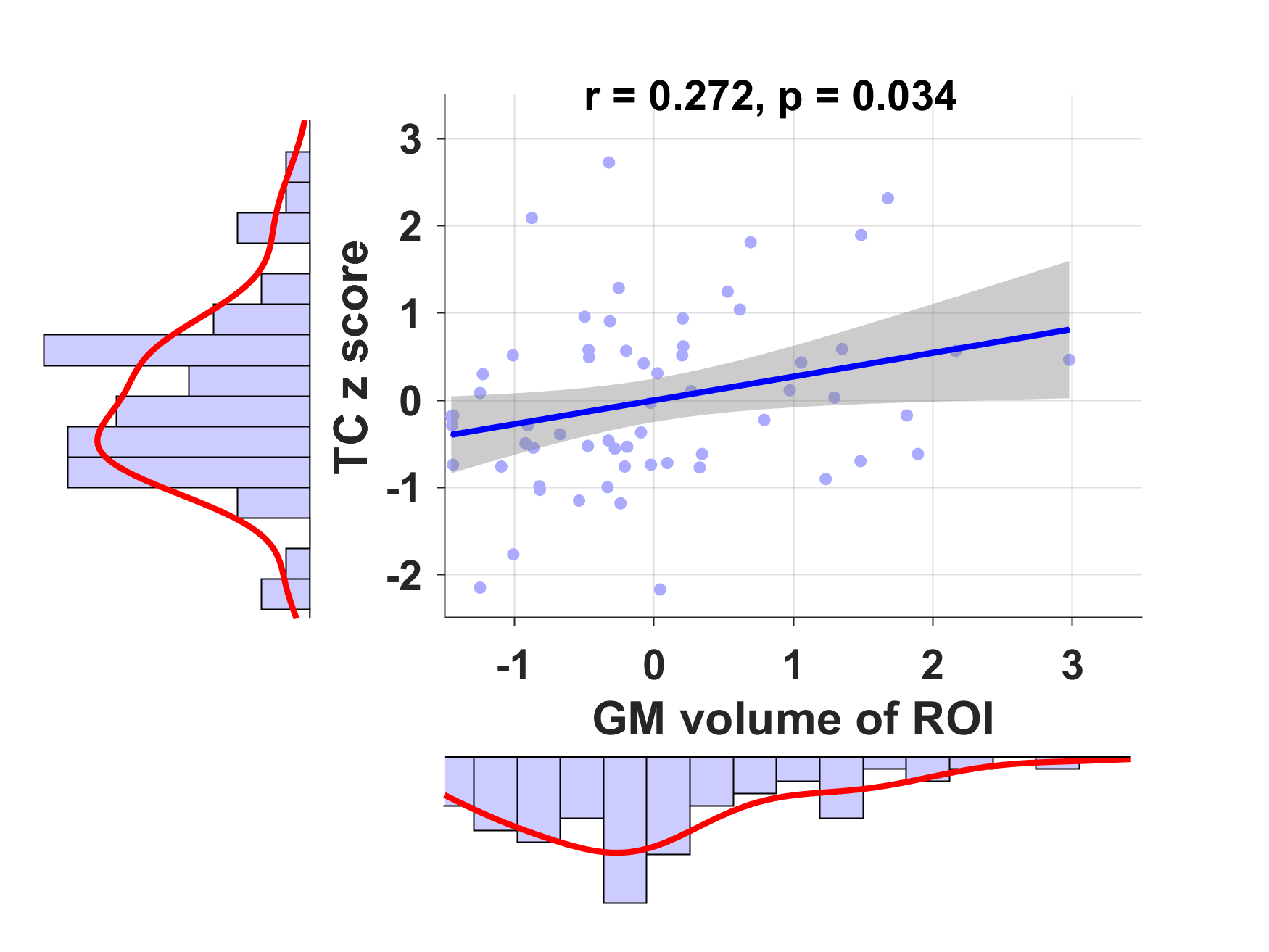

Supplement: Supplementary file 1 — Supporting Information [file BRB3-14-e70080-s002.zip › GMavg-TC.tif]

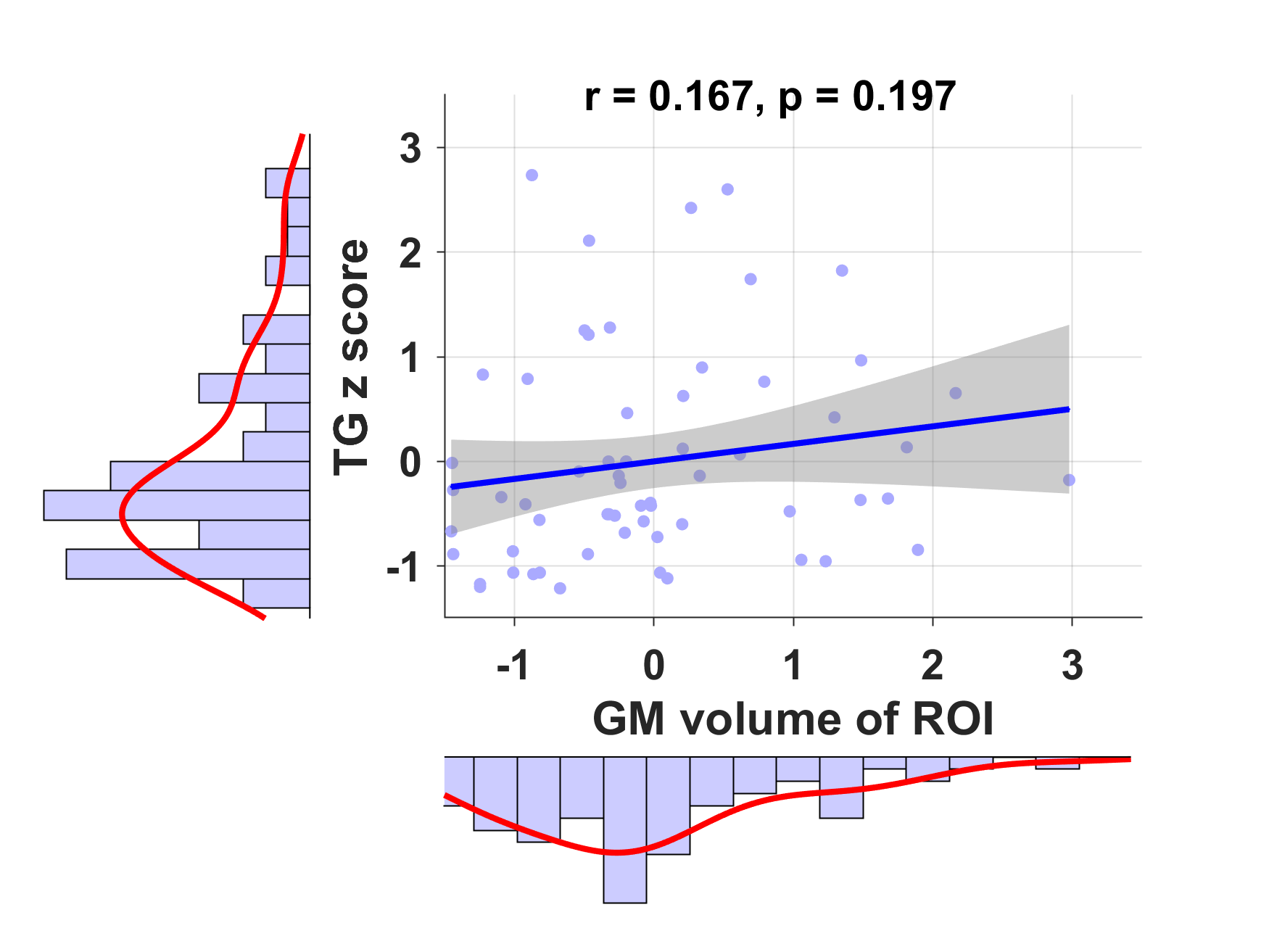

Supplement: Supplementary file 1 — Supporting Information [file BRB3-14-e70080-s002.zip › GMavg-TG.tif]

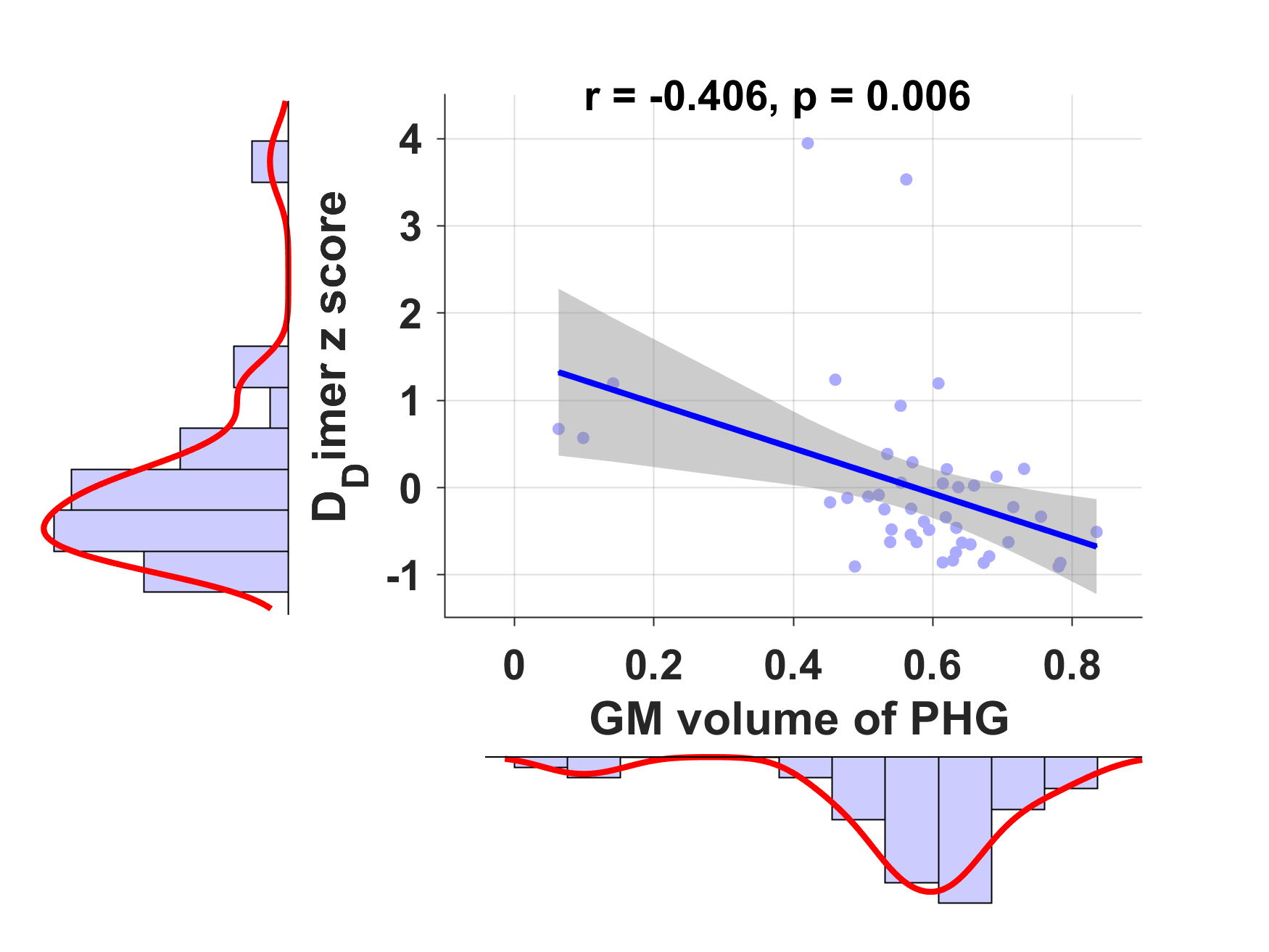

Supplement: Supplementary file 1 — Supporting Information [file BRB3-14-e70080-s002.zip › PHG GM-D_Dimer.tif]

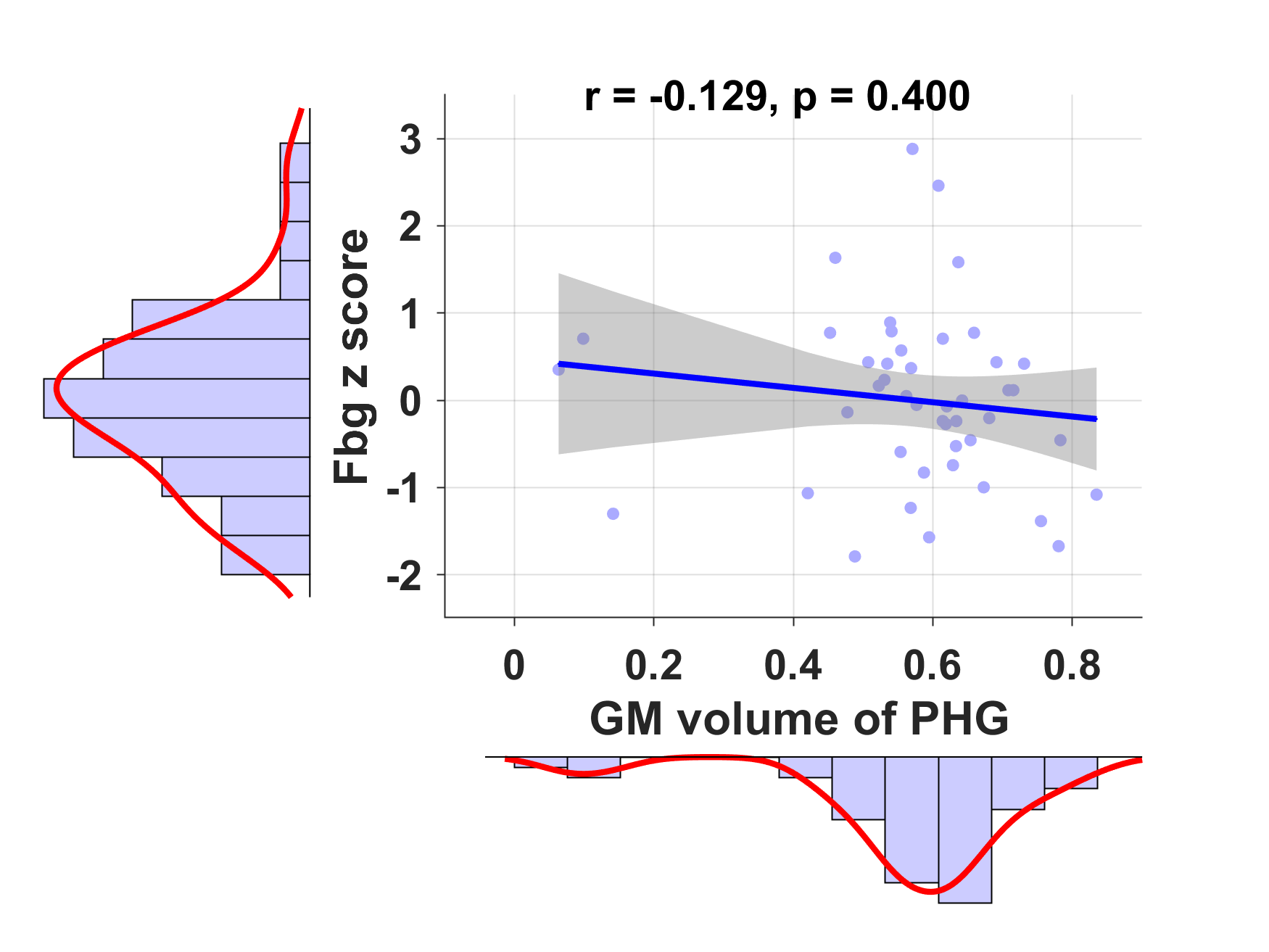

Supplement: Supplementary file 1 — Supporting Information [file BRB3-14-e70080-s002.zip › PHG GM-Fbg.tif]

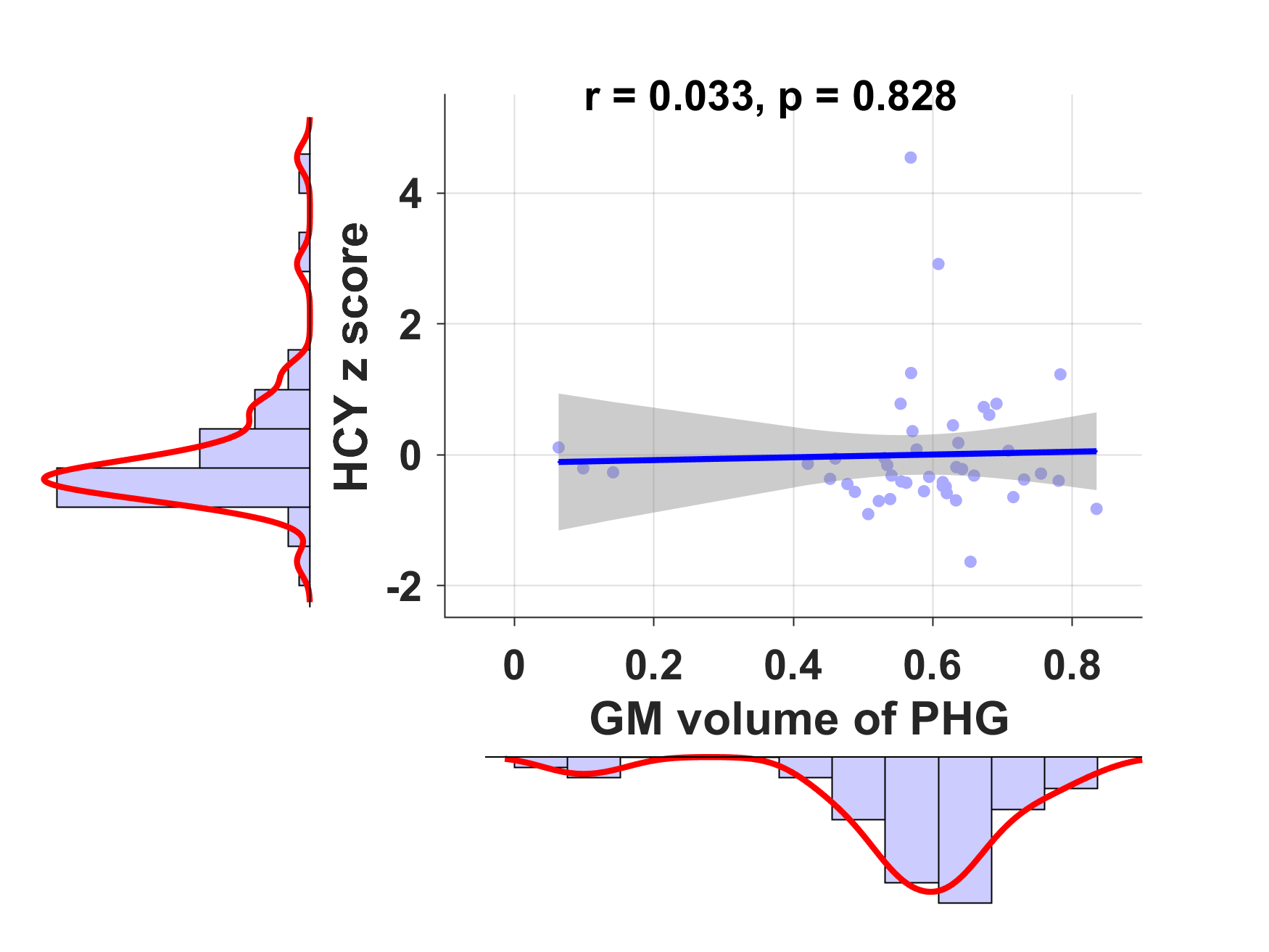

Supplement: Supplementary file 1 — Supporting Information [file BRB3-14-e70080-s002.zip › PHG GM-HCY.tif]

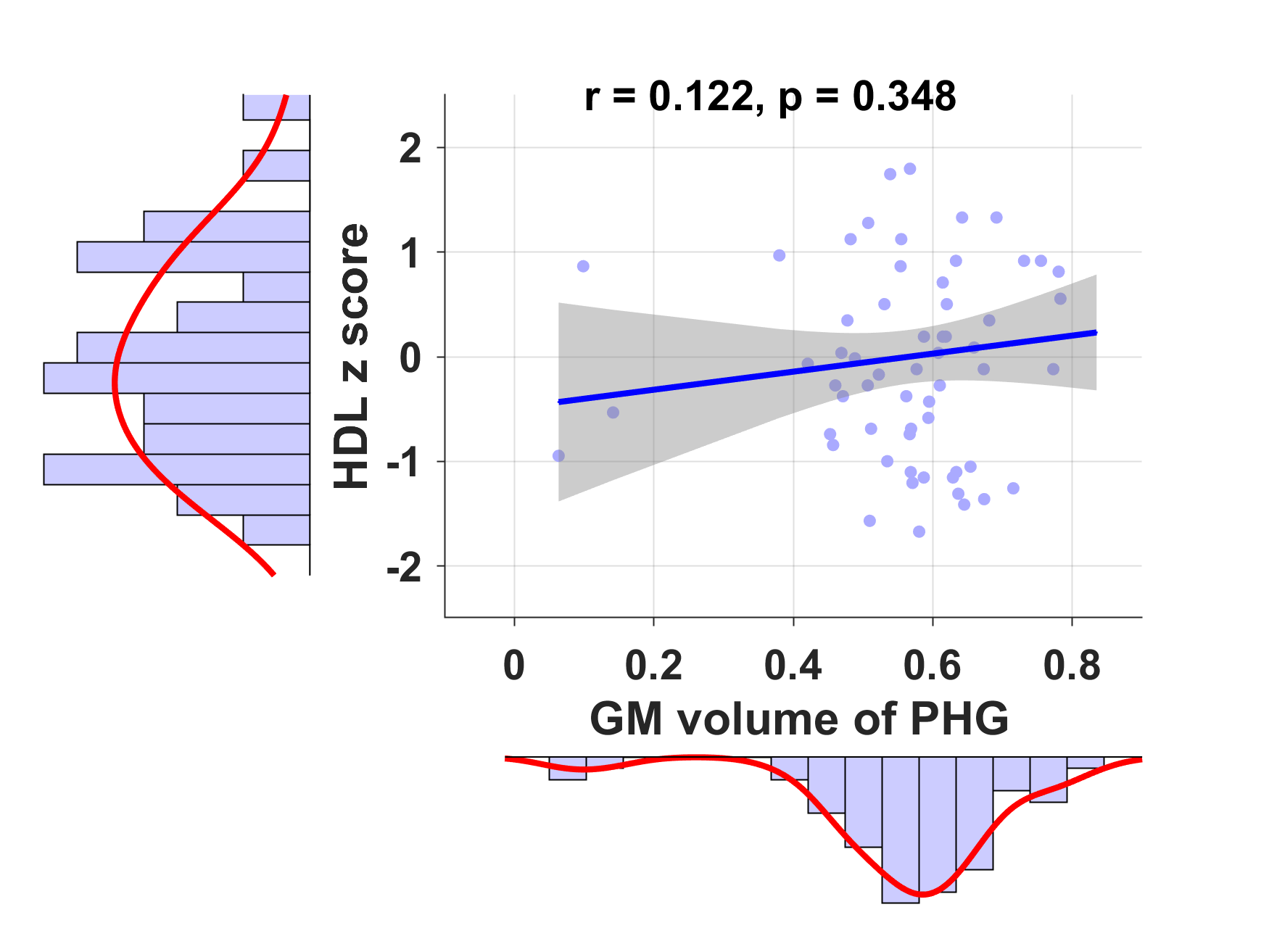

Supplement: Supplementary file 1 — Supporting Information [file BRB3-14-e70080-s002.zip › PHG GM-HDL.tif]

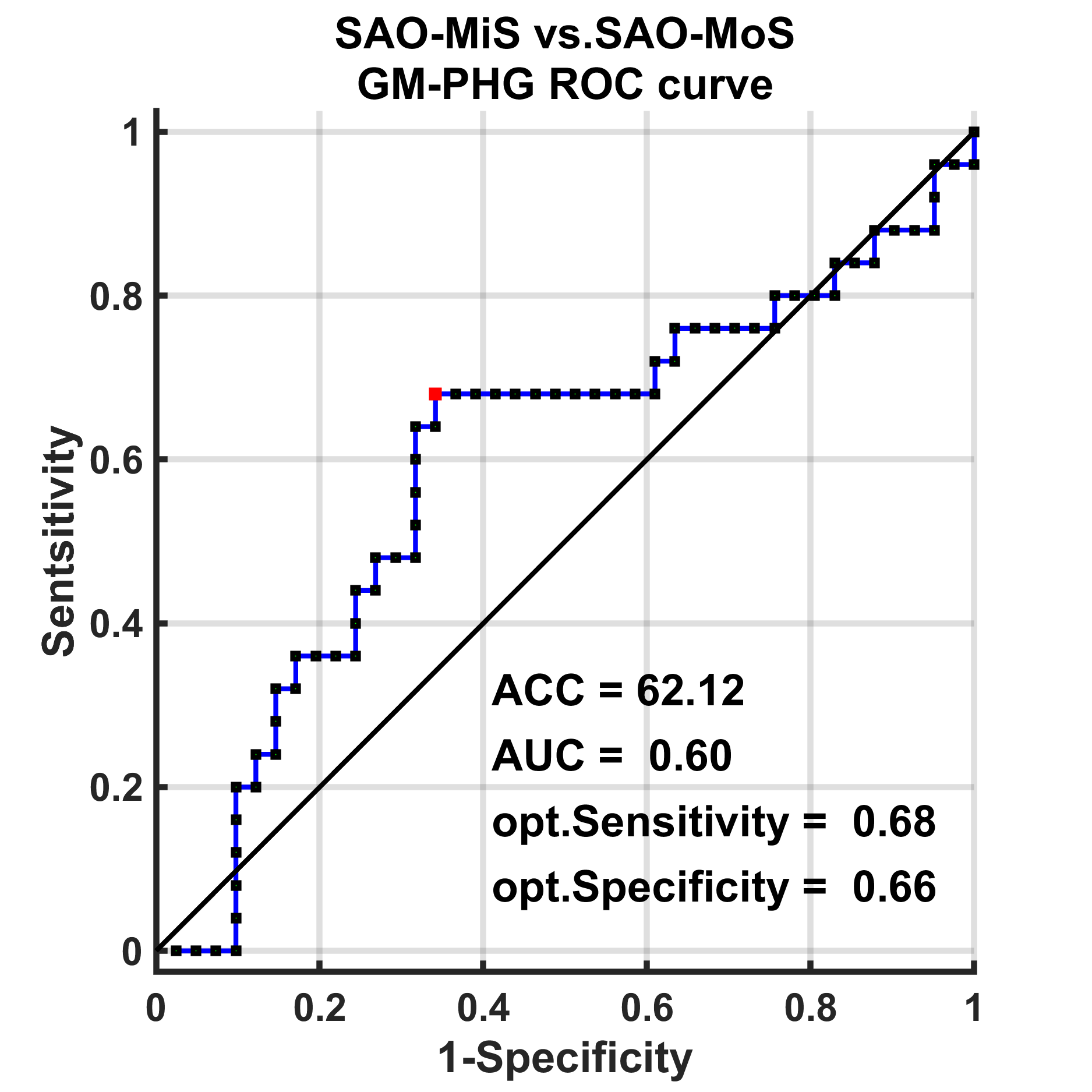

Supplement: Supplementary file 2 — Supporting Information [file BRB3-14-e70080-s001.zip › GM-PHG-SAO-MiS vs.SAO-MoS.tif]

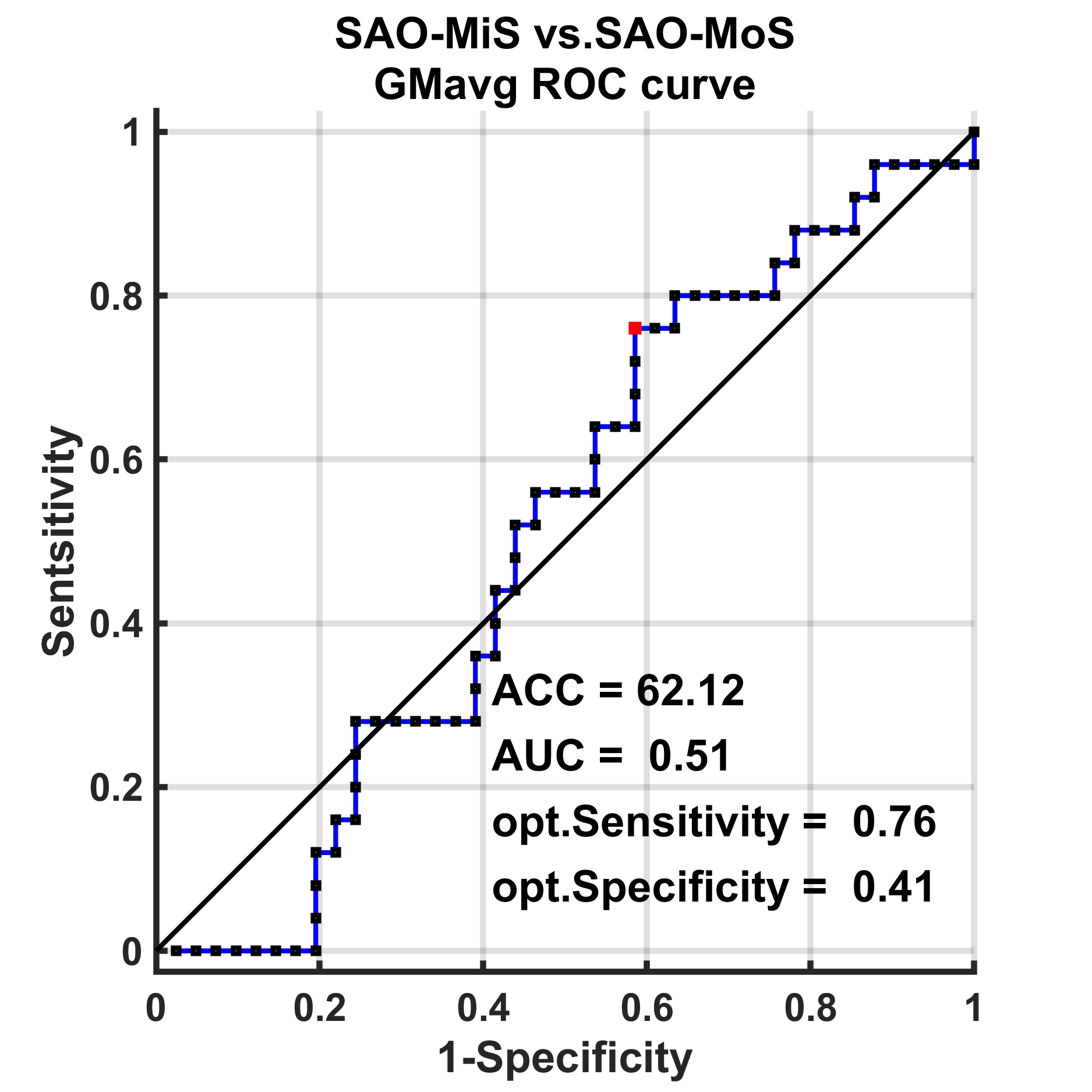

Supplement: Supplementary file 2 — Supporting Information [file BRB3-14-e70080-s001.zip › GMavg-SAO-MiS vs.SAO-MoS.tif]

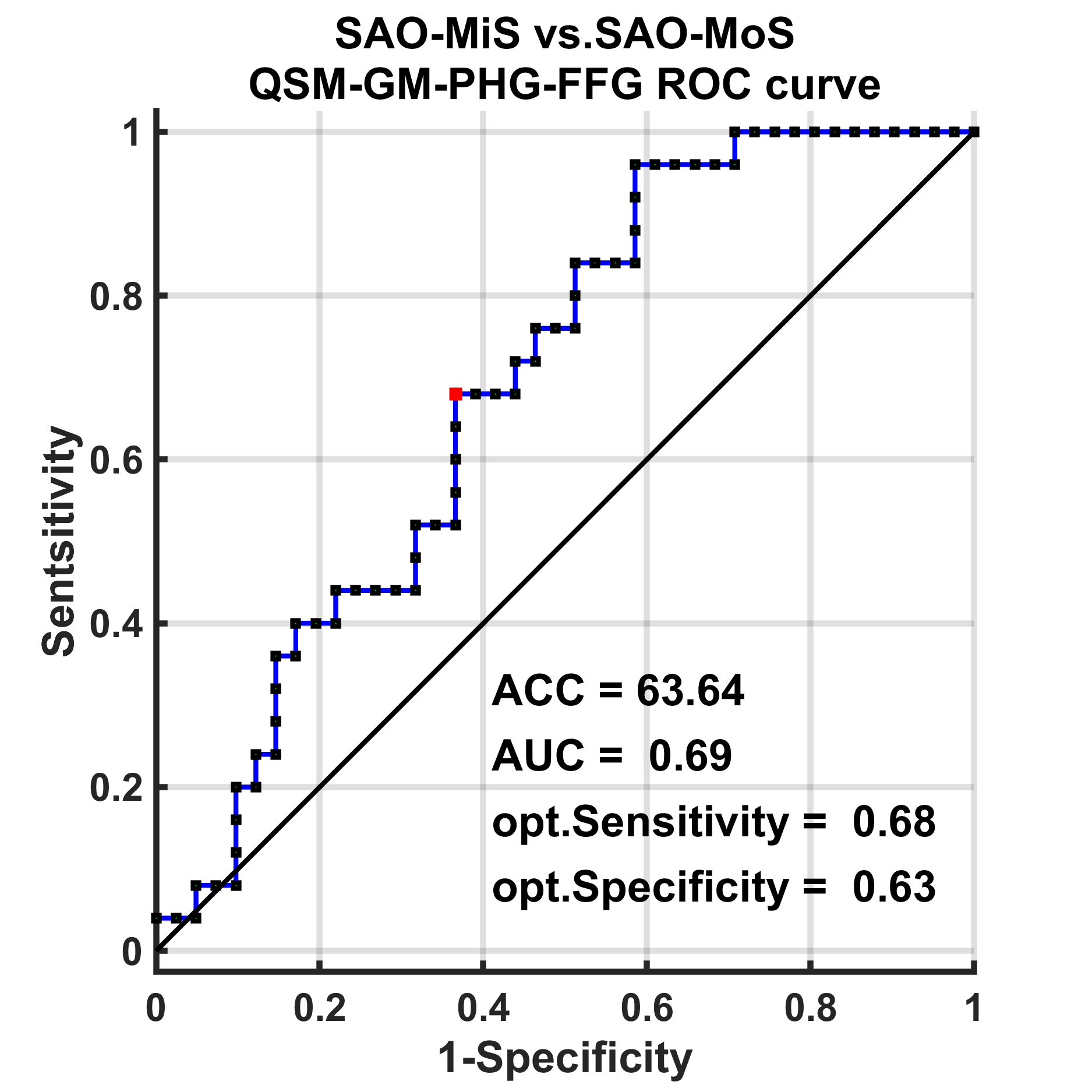

Supplement: Supplementary file 2 — Supporting Information [file BRB3-14-e70080-s001.zip › QSM-GM-PHG-FFG-SAO-MiS vs.SAO-MoS.tif]

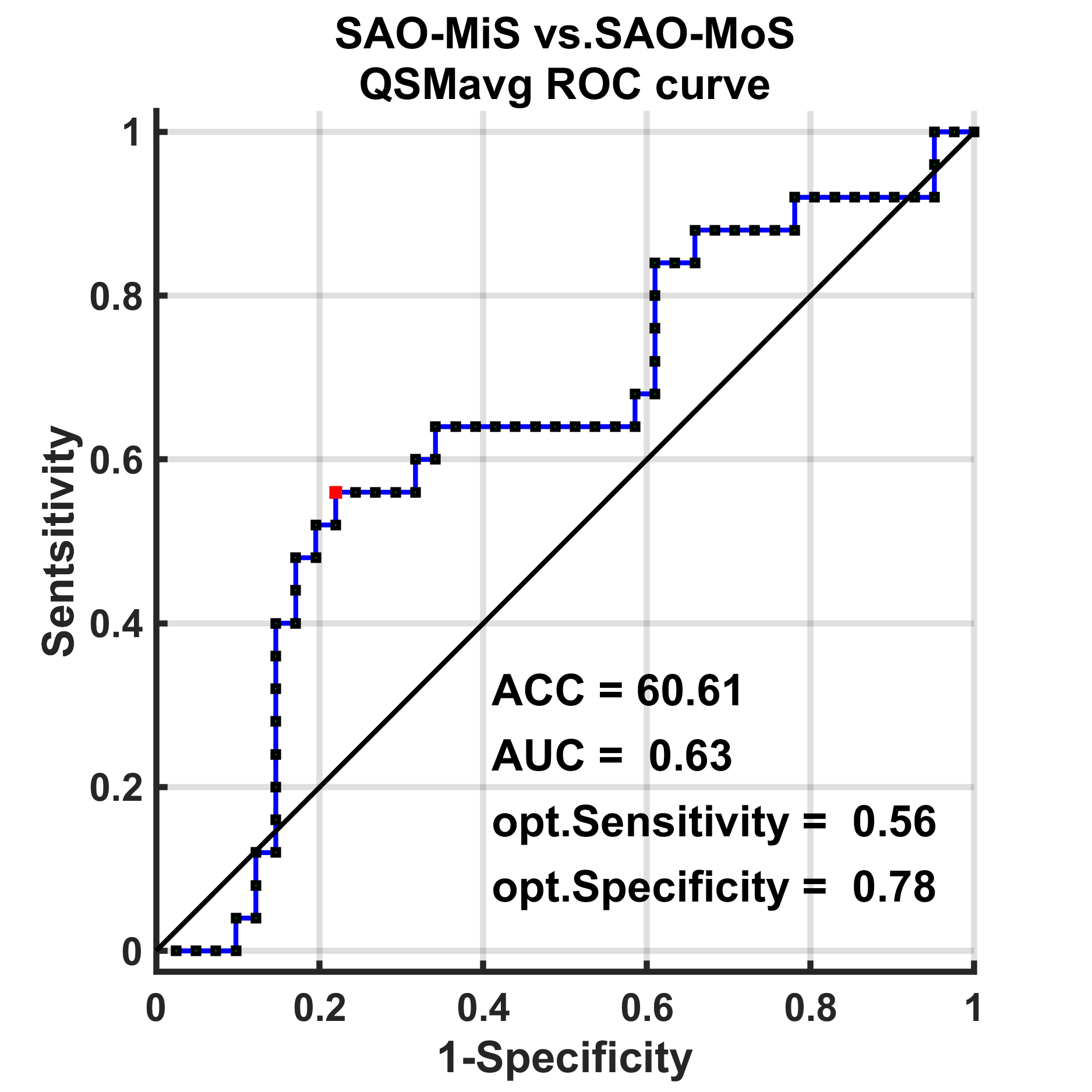

Supplement: Supplementary file 2 — Supporting Information [file BRB3-14-e70080-s001.zip › QSMavg-SAO-MiS vs.SAO-MoS.tif]

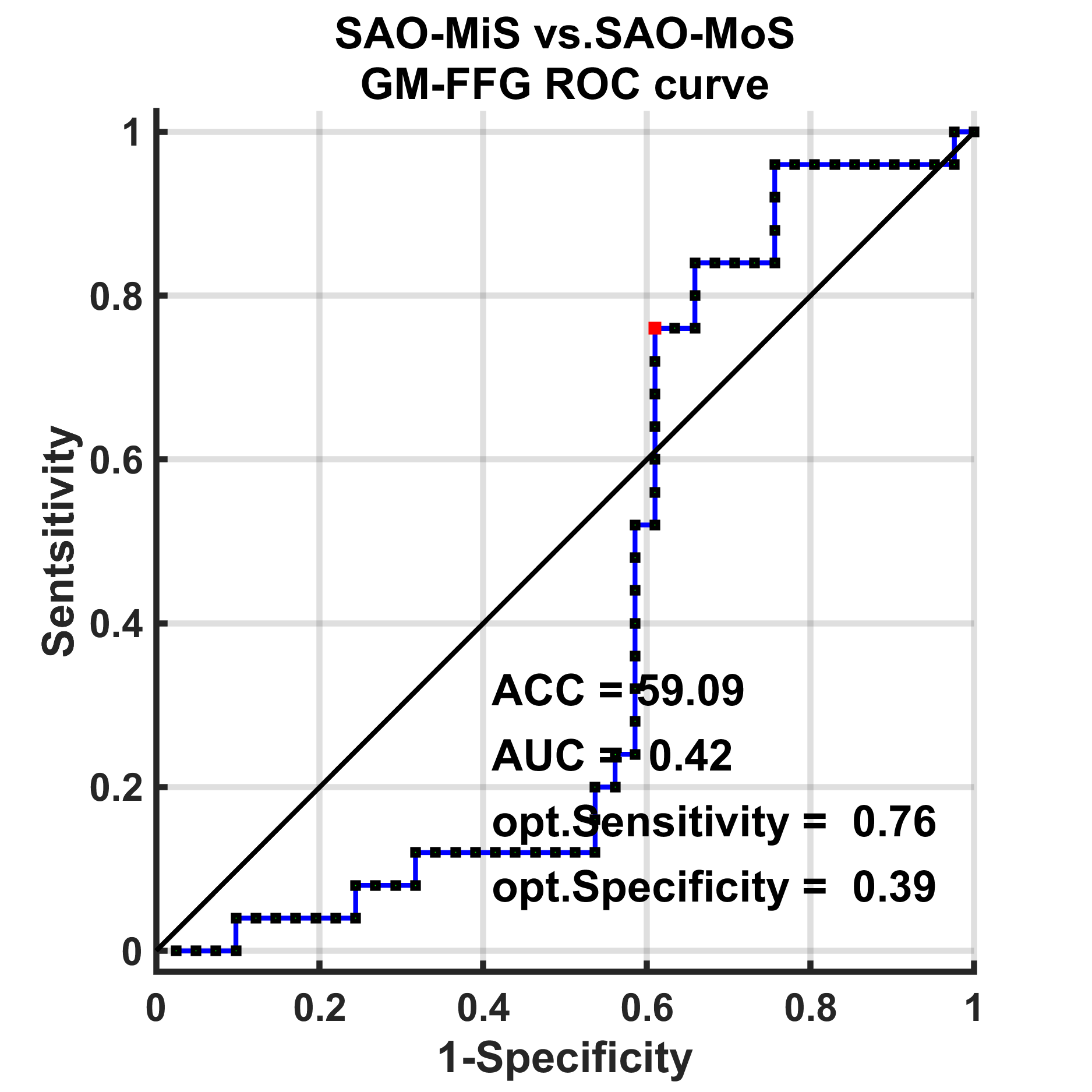

Supplement: Supplementary file 2 — Supporting Information [file BRB3-14-e70080-s001.zip › GM-FFG-SAO-MiS vs.SAO-MoS.tif]
